# Clinical and non-clinical aspects of reimbursement policy for orphan drugs in selected European countries

**DOI:** 10.3389/fphar.2024.1498386

**Published:** 2024-11-19

**Authors:** Szczepan Jakubowski, Przemysław Holko, Rafał Nowak, Marisa Warmuth, Marc Dooms, Outi Salminen, Lucas Cortial, Gisbert W. Selke, Christina Georgi, Einar Magnússon, Salvatore Crisafulli, Fons Strijbosch, Tanja Mueller, Eleanor Grieve, Immaculada Danés, Paweł Kawalec

**Affiliations:** ^1^ Department of Health Promotion and e-Health, Institute of Public Health, Faculty of Health Science, Jagiellonian University Medical College, Kraków, Poland; ^2^ Department of Nutrition and Drug Research, Institute of Public Health, Faculty of Health Science, Jagiellonian University Medical College, Kraków, Poland; ^3^ Department of Pharmaceutical Affairs, Federation of Social Insurances, Vienna, Austria; ^4^ University Hospitals Leuven, Leuven, Belgium; ^5^ Department for Steering of Healthcare and Social Welfare, Unit for Steering, Service Choices in Healthcare COHERE, Ministry of Social Affairs and Health, Helsinki, Finland; ^6^ Orphandev Fcrin Reference Network, Aix Marseille University, Assistance publique - Hopitaux de Marseille, Institut National de la Santé Et de la Recherche Médicale, Inst Neurosci Syst, CHU Timone, Marseille, France; ^7^ AOK Research Institute (WIdO), Berlin, Germany; ^8^ Health Economics and Management Lab, Department of Economic Science, University of Piraeus, Piraeus, Greece; ^9^ Pharmaceutical Affairs Ministry of Health, Reykjavík, Iceland; ^10^ Department of Medicine, University of Verona, Verona, Italy; ^11^ SiRM – Strategies in Regulated Markets, Utrecht, Netherlands; ^12^ Strathclyde Institute of Pharmacy and Biomedical Sciences, University of Strathclyde, Glasgow, United Kingdom; ^13^ Health Economics and Health Technology Assessment, School of Health and Wellbeing, College of Medical, Veterinary and Life Sciences, University of Glasgow, Glasgow, United Kingdom; ^14^ Clinical Pharmacology Department, Vall d’Hebron Hospital Universitari, Vall d’Hebron Barcelona Hospital Campus, Barcelona, Spain

**Keywords:** orphan drugs, rare diseases, EMA, clinical, policy, reimbursement, HTA, Europe

## Abstract

**Objectives:**

The aim of the study was to assess the reimbursement policy for orphan drugs (ODs) in selected European countries in relation to the availability and impact of clinical evidence, health technology assessment (HTA) procedures and reimbursement decision-making.

**Materials and Methods:**

A list of authorized ODs was extracted from a web-based registry of the European Medicines Agency, including information on active substance, Anatomical Therapeutic Chemical (ATC) classification code, and therapeutic area. A country-based questionnaire survey was conducted between September 2022 and September 2023 among selected experts from 12 European countries. A descriptive and statistical analysis was performed to identify correlations between country characteristic, HTA procedures, drug indication and positive recommendations or reimbursement decisions for ODs.

**Results:**

Safety assessment for ODs was mandatory in 10 countries, while it was optional in one country (Italy) and not required in one country (Iceland). Efficacy assessment for ODs was mandatory in 11 countries and not required in one country (Iceland). The impact of safety and efficacy assessment on reimbursement decisions was rated as high in 10 countries and as low in one country (Germany). Dedicated OD legislation and policies were reported in seven countries. In two countries (Belgium, Iceland), the HTA was not mandatory, and in one country (Germany), it only had an informative function. A positive recommendation (from an HTA agency or advisory body) guaranteed reimbursement in four countries, while a negative recommendation excluded reimbursement only in one country (Iceland). The proportion of reimbursed ODs ranged from 23.5% in Iceland to 86% in Germany (*p* < 0.001). ODs with ATC code L represented the largest group of medicines (n = 49). They were also very frequently reimbursed ODs in the countries studied, with a mean of 61.8% (*p* < 0.001).

**Conclusion:**

European countries differ in terms of the impact of clinical issues and additional clinical aspects on the reimbursement policy for ODs. Reimbursement decisions were affected by OD-specific legislation, policies, and EMA authorization status. HTA dossiers and procedures significantly influenced reimbursement decisions, although some ODs were reimbursed regardless of the positive or negative recommendations. ATC codes were significantly correlated with reimbursement status and positive recommendation.

## 1 Introduction

Looking at the drug therapies available in Europe, we can see that some drugs are more commonly used than others (Howie et al., 2013). One of the key factors that can explain this variability is the number of patients in Europe with a particular disease that can be treated with drug therapies. This means that some therapeutic areas may be more “attractive” to the pharmaceutical industry than others. Before a drug is approved and made available to patients, it has to go through a long and expensive process (Howie et al., 2013).

Initially, research is conducted to determine whether a given compound is promising enough to be evaluated in a preclinical trial, the final step before the potential medicinal product is first used in humans. The product then undergoes three phases of clinical trials (with some exceptions) to confirm its safety and efficacy and to determine the dosing regimen for the best therapeutic outcome. Finally, once safety and efficacy are confirmed, the drug can be approved for use, which typically takes years and is very costly. The cost of developing an orphan drug (OD) was on average higher than non-ODs (Marino et al., 2023). This means that pharmaceutical companies will carefully select areas of interest for drug development. The cost of developing a new drug must be recouped in a reasonable period of time. Thus, the expected uptake of a drug must be considered when calculating its final price.

Drugs for rare diseases are referred to as ODs or orphan medicinal products. According to the European Commission, ODs are intended for the diagnosis, prevention, or treatment of life-threatening or very serious conditions that affect no more than 5 in 10,000 people in the European Union (EU) (European Commission, 2023).

To encourage the development of ODs, various governments around the world have established regulatory frameworks and incentives. For example, in the United States, the Orphan Drug Act of 1983 provides tax credits, grants, and market exclusivity to encourage pharmaceutical companies to invest in the research and production of ODs (U.S. Food and Drug Administration, 2024). Similarly, the European Medicines Agency (EMA) offers incentives to OD manufacturers, such as fee reductions and protocol assistance. Manufacturers wishing to benefit from these incentives have to apply to the EMA for orphan designation. This is to ensure that the drug being developed falls within the scope of ODs. In the EU, a centralized procedure is used to evaluate the marketing authorization application of a designated OD. In some cases, these drugs are made available to patients under conditional marketing authorization (European Medicines Agency, 2024a).

Another consideration for each EU Member State is the impact on its healthcare budget if all eligible patients enrolled in clinical trials receive the therapy. Standard health technology assessment (HTA) can inform reimbursement decisions. Understanding the HTA criteria for rare diseases that have been implemented in different public health contexts is therefore essential to enrich the discourse and potentially contribute to the creation of more equitable and successful evidence-based policies that address the difficulties associated with access to ODs (Felippini et al., 2024). HTA dossier, market availability, regulatory approval, governmental incentives for research, and reimbursement mechanisms all play an important role in the complicated process of gaining access to ODs (Detiček et al., 2018).

The reimbursement of ODs is frequently determined by the exceptions set out in publicly funded healthcare systems (Zimmermann et al., 2021). It is problematic from a clinical perspective, as it places the responsibility for determining whether and when to reimburse ODs on clinicians and healthcare insurers. (Zimmermann et al., 2021). This approach raises equity and fairness concerns in terms of access to these drugs. If the current scientific discourse were to place greater emphasis on the crucial variabilities of different ODs regarding target populations, cost-effectiveness, the level of evidence, or the mechanism of action in HTA, it might prove instrumental in resolving the issue of OD reimbursement (Zimmermann et al., 2021). In addition, some studies have indicated that lower-quality clinical evidence, a higher level of uncertainty regarding the clinical efficacy and safety of ODs, is still accepted for submission for reimbursement (Dupont and Van Wilder, 2011).

The above brief overview of ODs and their possible routes of entry into the market indicates that it can be challenging for patients from EU Member States to achieve equitable access to these therapies.

The aim of the study was to assess the reimbursement policy for ODs in selected European countries in relation to the availability and impact of clinical evidence, selected aspects (additional clinical determinants, regulatory aspects, EMA authorization status) and HTA in context of reimbursement decisions making. Furthermore, the study attempts to identify the drug type according to indication and other factors that contribute to the discrepancy between the number of drugs with a positive recommendation from HTA agency/advisory bodies and the number of drugs that have received reimbursement. We also discussed the mechanisms of OD reimbursement policy (including HTA) and highlighted the specificities of the OD sector.

## 2 Materials and methods

In the preliminary phase of the study, in September 2022, a list of authorized medicines with orphan designation was obtained from a web-based registry of the EMA, including information on the active substance, the Anatomical Therapeutic Chemical (ATC) classification code, and the therapeutic area (European Medicines Agency, 2024b).

To obtain detailed information on reimbursement policies for ODs, a questionnaire survey was conducted among experts in reimbursement and drug market access from selected European countries. The objective was to collect the most up-to-dated and comprehensive data so we invited to take part in our survey the most appropriate experts from specified countries to collect such input. The survey was performed by e-mail in period from September 2022 to September 2023, with 2022 as the year under study. Experienced experts who have coauthored scientific publications on OD reimbursement or market access available in Medline and Google Scholar were invited. The criteria for the selection of experts were as follows: a proven expertise in OD reimbursement or OD market access, and practical experience in related fields. Finally, selected experts were only accepted if they declared no conflict of interest in relation to the study, as determined by a review of their published work.

The invitation was accepted by 13 experts from twelve European countries: Austria (number of included experts; n = 1); Belgium (n = 1); Finland (n = 1); France (n = 1); Germany (n = 1); Greece (n = 1); Iceland (n = 1); Italy (n = 1); Netherlands (n = 1); Scotland (n = 2) and Spain (n = 1); Poland (n = 1), which is the biggest and the most populated country of Central Eastern Europe, was also included as a reference. In the case of Scotland, two experts had to be employed to collect all relevant information. The background of the experts was as follows: 8 people represented academia; 2 persons national health authority; 1 person was affiliated with a national health insurance institution; 1 expert with an healthcare consulting organization and 1 expert with research organization.

The questionnaire (created for purposes of the study) explained the research objective and included open and closed questions. The questionnaire was validated by selected expert, then revised and improved based on the results, and double-checked by the authors. The survey consisted of a total of 37 questions on the ODs reimbursement policy. A set of questions assessed the following aspects related to ODs: 1) aspects (clinical and additional) considered in reimbursement decisions; 2) pharmaceutical policy mechanisms and strategies; 3) the use of HTA dossiers in the reimbursement process; and 4) recommendations and reimbursement status of the medicines.

Based on the qualitative analysis of the questionnaires, descriptive country profiles of reimbursement and HTA process for ODs were developed. An analysis of safety, efficacy, and other aspects (additional clinical determinants, regulatory context, EMA authorization status) of ODs in the reimbursement decision has been carried out. Information on the recommendation and reimbursement decision-making and implementation of HTA dossiers was also included. Data on the number of ODs with a positive recommendation and the number of ODs reimbursed in each country were analyzed descriptively and presented as frequencies and percentages. The analyses were performed separately for the subgroups of conditions classified by the anatomic main group of the ATC code. The χ^2^ Pearson test was used to compare OD status between countries (i.e., reimbursed, or non-reimbursed OD; OD with or without a positive recommendation for reimbursement). A series of logistic regression models with nested random effects (a drug variable within a country variable) and a single fixed effect model were performed to identify the country or drug characteristics that may be associated with the reimbursement status or a positive recommendation for an OD across selected European countries. A *p*-value of less than 0.05 was considered significant. Data were prepared and analyzed using Stata 17SE (StataCorp., College Station, TX, United States) and OriginPro 2021b (OriginLab Corporation, Northampton, MA, United States).

## 3 Results

### 3.1 Country-specific characteristics: clinical aspects of ODs in reimbursement decision making, reimbursement policy, and HTA procedures for ODs in selected European countries

Based on the results presented in Table 1, safety assessment was mandatory in 10 countries, optional in one country (Italy) and not mandatory in one country (Iceland). Efficacy assessment was mandatory in 11 countries, while it was not required in one country (Iceland). In Iceland, reimbursement decisions were based on safety and efficacy assessments from other countries. The impact of safety and efficacy assessment on reimbursement decisions was rated as high in 10 countries and as low in one country (Germany). An acceptable safety profile had to be defined in all the countries studied, except for Belgium, while the definition of an acceptable efficacy profile was used in almost half of the countries (n = 5). It was more common to reimburse an OD with insufficient evidence on efficacy than on safety (5 vs. 1). Separate criteria for assessing safety (n = 2) and efficacy (n = 0) for pediatric populations were rarely required. In almost all countries (n = 10), other clinical aspects (e.g., severity and burden of disease, tolerability of the substance) influenced reimbursement decision making. Dedicated OD legislation and policies were identified in seven countries. The EMA appears to have a significant impact on reimbursement decisions in the countries studied: in eight countries, reimbursement decisions were influenced by the authorization status or orphan designation granted by the EMA.

**TABLE 1:** Safety, efficacy, and other aspects of orphan drugs in the reimbursement process in selected European countries.

Characteristics	Austria	Belgium	Finland	France	Germany	Greece	Iceland	Italy	Netherlands	Poland	Scotland	Spain
Safety assessment [not required, voluntary, mandatory]	mandatory	mandatory	mandatory	mandatory	mandatory	mandatory	not required	Voluntary^d^	mandatory	mandatory	Mandatory^e^	mandatory
Definition of acceptable safety profile	no	yes	no	no	no	no	no	no	no	no	no	no
Reimbursement without sufficient evidence of safety	partially	no	no	no	n/a^a^	no	n/a^c^	Yes^d^	no	no	n/a^e^	no
Similar safety profile to pediatric and adult assessment	yes	yes	yes	yes	yes	yes	yes	n/a^d^	no	yes	yes	no
Efficacy assessment [not required, voluntary, mandatory]	mandatory	mandatory	mandatory	mandatory	mandatory	mandatory	not required	mandatory	mandatory	mandatory	mandatory	mandatory
Definition of acceptable efficacy profile	yes	no	yes	no	no	n/a^b^	no	yes	yes	no	yes	no
Reimbursement without sufficient evidence of efficacy	yes	no	no	partially	n/a^a^	yes	n/a^c^	yes	no	no	yes	yes
Similar safety profile to pediatric and adult assessment	yes	yes	yes	yes	yes	n/a^b^	yes	yes	yes	yes	yes	yes
Impact of safety and efficacy on reimbursement decisions [low, moderate, high]	high	high	high	high	low	high	n/a^c^	high	high	high	high	high
Other clinical aspects influencing reimbursement process	yes	yes	yes	yes	yes	yes	n/a^c^	no	yes	yes	yes	yes
Dedicated to ODs legislation and policies	no	yes	no	no	yes	yes	no	yes	yes	yes	yes	no
Reimbursement decisions dependent on EMA authorization status and ODs designation	yes	yes	no	no	yes	yes	yes	yes	no	no	yes	yes

EMA, European Medicines Agency | ODs, Orphan drugs |n/a - not applicable.

^a^ In Germany, EMA-authorized ODs, were considered sufficient safe and effective to be reimbursement.

^b^ In Greece, the physician may request reimbursement of an unavailable/unapproved medicine for the individual patient (adult, child).

^c^ In Iceland, safety and efficacy assessments of ODs, were not obligatory and decision based on results from other Nordic countries (Denmark, Norway, Sweden, Finland).

^d^ In Italy, safety assessment was optional and legislative instruments played a significant role in access to medicines.

^e^ In Scotland, analysis made by the EMA (during authorization) were taken as sufficient in safety assessment.

Based on the results presented in Table 2, the HTA was not mandatory in only two countries (Belgium, Iceland) and it had an informative function in one country (Germany). Almost all countries had a dedicated advisory body (n = 10). A positive recommendation (from an HTA agency or advisory body) guaranteed reimbursement in four countries (Finland, France, Greece, and Iceland), while a negative recommendation excluded reimbursement in only one country (Iceland). Increasing the willingness-to-pay threshold (for cost per quality-adjusted life years) for ODs relative to non-ODs was not a common practice. Specific cost-utility thresholds were used in only one country (Scotland), but they were set individually for the OD. The most common HTA analysis for ODs in the countries studied was cost-effectiveness analysis (n = 10), while cost-benefit analysis (n = 2) was the least common.

**TABLE 2:** Recommendation and HTA analyses of orphan drugs in selected European countries.

Characteristics	Austria	Belgium	Finland	France	Germany	Greece	Iceland	Italy	Netherlands	Poland	Scotland	Spain
Obligatory HTA	yes	no	yes	yes	Yes^a^	yes	no	yes	yes	yes	yes	yes^b^
Institution applying for reimbursement	MAH	MAH	MAH, patients, or unlicensed medicines importer	MAH	MAH	MAH	MAH	MAH	MAH	MAH	MAH	MAH
Special advisory institution/-s	yes	yes	no	yes	yes	yes	yes	yes	yes	yes	yes	no
Positive recommendation (from HTA agency/advisory body) ensures reimbursement	no	no	yes	yes	no	yes	yes	no	no	no	no	no
Negative recommendation (from HTA agency/advisory body) excludes reimbursement	no	no	partially	no	no	no	yes	no	no	no	no	no
ICER/ICUR thresholds the same for ODs as for non-ODs	n/a	yes	yes	n/a	n/a	n/a	n/a	yes	yes	yes	no no fixed thresholds framework	n/a
HTA ANALYSES REQUIRED IN A REIMBURSEMENT PROCESS												
Benefit-risk analysis	+^c^	+	+	+	−	−	−	−	−	−	+	+
Budget impact analysis	+^c^	+	−	−	−	+	−	+	+	+	+	+^d^
Clinical analysis	+^c^	+	−	+	+	+	−	+	−	+	+	+
Cost−benefit analysis	+^c^	+	−	−	−	−	−	−	−	−	−	−
Cost−effectiveness analysis	+^c^	+	+	+	−	+	−	+	+	+	+	+^d^
Cost−minimization analysis	+^c^	+	−	−	−	−	−	−	−	+^e^	−	−
Cost−utility analysis	+^c^	+	+	+	−	+	−	+	+	+	+	−

HTA, Health Technology Assessment | MAH, Marketing Authorization Holder | ICER, Incremental Cost-Effectiveness Ratio | ICUR, Incremental Cost Utility Ratio | ODs, Orphan drugs, n/a - not applicable.

^a^ Only informative function, because country-level HTA, is not required for ODs, in Germany.

^b^ There is a health echnology assessment but there is no one national Health Technology Assessment agency in Spain.

^c^ It could be any of these analysis depending on the claim of and documents submitted by the MAH.

^d^ not included in the HTA, dossier.

^e^ included in rare situations.

In addition to the results presented in Tables 1, 2, detailed country-specific characteristics of clinical and other aspects considered in reimbursement decisions, reimbursement policies, and HTA procedures for ODs are described below.

#### 3.1.1 Austria

In Austria, the marketing authorization holder (MAH) submits an application for a drug to be included in the positive list of outpatient drugs (in German, Erstattungskodex [EKO]) reimbursed by the Austrian Social Insurance Institutions (in German, Sozialversicherungsträger). Next, the Department of Pharmaceutical Affairs at the Federation of Social Insurances (in German, Dachverband der Sozialversicherungsträger [DVSV]), an umbrella organization of the Social Insurance Institutions prepares both a pharmacological/medical-therapeutic and a health economic evaluation (in comparison with existing therapeutic alternatives).

In Austria, safety and efficacy assessment have a high impact on the reimbursement decisions, but for some drugs factors such as unmet medical need or the lack of therapeutic alternatives are also taken into account. Aspects considered in the safety assessment of ODs (like for other drugs) includes overall adverse events (AEs), treatment discontinuation due to AEs, serious AEs, deaths, and other AEs of special interest (disease- and/or drug-specific).

A new drug may be reimbursed through the reimbursement procedure, if it is at least as good as or similar to a therapeutic alternative; if it is worse than the existing therapeutic alternative, the new drug would in general not always be reimbursed. A substantial additional therapeutic benefit (for a subgroup or the majority of patients) compared with existing therapeutic alternatives is defined by several factors, including significant improvements (at least by recognized surrogate endpoints), such as – depending on the disease – reduction of symptoms (significantly faster and/or more complete), improved survival, avoidance or delay of sequelae, or absence of severe side effects; for chronic diseases, it is a significant (clearly objectifiable) improvement in the quality of life. This applies to both pediatric and adult drugs, including ODs. There are exceptions where an acceptable efficacy profile for the OD cannot be proven but in the overall decision the drug is still accepted for reimbursement (for a defined time period until re-evaluation). These are cases where efficacy may be assessed as acceptable based on surrogate rather than patient-relevant endpoints or where at least general (pharmacological) efficacy has to be demonstrated (e.g., antitumor activity in phase I and II trials). The efficacy assessment addresses some aspects related to the credibility of clinical trials, including the type of the trial (criteria: controlled trials for efficacy and safety assessment, uncontrolled trials for safety assessment only), the phase of the trial (preferably phase III trials, but if unavailable, also phase II trials). In general, the risk of bias is not systematically assessed.

The health economic evaluation is based on the pharmacological/medical-therapeutic evaluation and takes into account, e.g., the price of therapeutic alternatives, the therapeutic value of the drug to be included compared to existing therapeutic alternatives listed in the EKO, and the European average price. Finally, if the medicine has a substantial additional therapeutic benefit or there is no therapeutic alternative on the reimbursement list, the MAH is required to submit a pharmacoeconomic study (preferably a cost-utility analysis) to demonstrate an appropriate cost-benefit ratio.

The reimbursement decision by DVSV is based on a recommendation by an advisory body for outpatient drug reimbursement decisions, the Drug Evaluation Committee (in German, Heilmittel-Evaluierungs-Kommission [HEK]). The appraisal of the evaluation of medicines for inclusion into the EKO is conducted by HEK on a monthly basis. Theoretically, DVSV can include drugs in the EKO even if HEK does not recommend reimbursement, and *vice versa*. However, DVSV usually follows the recommendation of HEK.

If inclusion into the EKO is not recommended by HEK and/or rejected by DVSV, the drug may still be reimbursed on a case-by-case basis for individual patients in justified exceptional cases (e.g., if there is no therapeutic alternative available) after prior approval by the medical officers of the Social Insurance Institutions.

In Austria, reimbursement decisions for inpatient drugs (e.g., determined by the route of administration or the need for specific infrastructure) lies within the remit of the hospitals and the nine federal states of Austria, respectively, and hence do not follow the process described for outpatient drugs.

#### 3.1.2 Belgium

In Belgium, there was a specific legislation and policy for ODs. The central point was a programme entitled the National Plan Rare Diseases (in Dutch, Belgisch plan voor Zeldzame Ziekten) from 2013. This Plan continues to operate today without major changes – the only change (article 81/111) is that access has been granted to innovative ODs if the pharmaceutical companies support “real world data” (data from patient treatment in practice) follow-up in these patients. The (interim) reimbursement contracts for innovative ODs were made available in a web application (www.webappsa.riziv-inami.fgov.be) for transparency purposes, providing easy access to the non-confidential parts of the contracts and annexes. Orphan drugs had to show an acceptable safety profile to be reimbursed, and the acceptable profile has been defined as a drug with non-serious side effects. The safety assessment of ODs was based on all aspects addressed in the EMA reports and medical literature, while the efficacy assessment was mainly based on the analysis of randomized controlled trials. In addition to the general safety and efficacy assessment, other clinical aspects that influence reimbursement decisions included QoL and compounding (magistral preparations) – medicinal products prepared in a pharmacy for a specific patient in compliance with a doctor’s prescription. Moreover, all reimbursed ODs were dispensed in hospitals. HTA was not mandatory for ODs, but there was the Belgian Healthcare Knowledge Centre (KCE), which acted as an advisory body for drug reimbursement. The MAH was the only institution requesting reimbursement for the drug. If an ODs was granted reimbursement it was 100%.There were no formal restrictions on submitting other analyses in addition to the primary HTA analyses (Table 2).

#### 3.1.3 Finland

In Finland, the safety assessment of ODs was most often based on aspects such as published evidence on clinical safety and risk-to-benefit ratio. Orphan drugs had to show an acceptable efficacy profile to be reimbursed, and the acceptable profile was defined as a drug with significant therapeutic value. The efficacy assessment of ODs was based on the evaluation of the validity and generalizability of clinical trials. In addition to the general safety and efficacy assessment, other clinical aspects influencing reimbursement decisions included sufficient and overall therapeutic benefit. Basically, the principles of evaluation were similar for ODs and other medicines. HTA was mandatory for ODs, but there was no advisory body for drug reimbursement – all recommendations and reimbursement decisions for outpatient medicines were provided by the Pharmaceuticals Pricing Board (in Finnish, Lääkkeiden Hintalautakun [HILA]). In special situations, other institutions such as the Finnish Medicines Agency and the Council for Choices in Healthcare in Finland (in Finnish, Terveydenhuollon Palveluvalikoimaneuvosto [PALKO]), were authorized to perform HTA and issue recommendations for drugs intended for use in hospitals. An OD with a positive recommendation from HILA was guaranteed reimbursement, while PALKO recommendations were considered by hospitals but were not mandatory. Outpatient medicines could not be reimbursed if they had a negative recommendation, while hospitals could choose to fund treatments not recommended by PALKO for a single patient or for a defined, usually small, group of patients (so called mini-HTA).

#### 3.1.4 France

In France, the reimbursement evaluation process was the same for ODs and non-ODs. Reimbursement was granted or withheld according to the actual benefit (in French, service médical rendu [SMR]), assessed by the Transparency Committee (Commission de la Transparence [CT]) of the French Health Authority (Haute Autorité de Santé [HAS]). The SMR, which can be either important, moderate, weak, or insufficient, was assessed according to five main criteria: 1) efficacy and adverse effects; 2) substitution with other therapy (comparators); 3) disease severity; 4) preventive, curative or symptomatic effect of the drug; and 5) the public health benefits of the drug (e.g., mortality, morbidity, improvement in QoL and disabilities avoided). In addition, CT measured the clinical added value with the degree of “improvement in actual benefit” (in French, amélioration du service médical rendu [ASMR]) ranging from ASMR 5, indicating no improvement, to ASMR 1, indicating major improvement. ASMR had a great impact on the reimbursement level, as discussed with the Economic Committee for Health Products (in French, Comité Economique des Produits de Santé). On 1 July 2021, HAS introduced an “early access program,” making innovative drugs (including ODs) available to patients free of charge. In February 2023, the program was slightly updated to ensure relevant HTA. The OD safety assessment was specific to each drug and depended on the risk-benefit ratio. The safety profile was assessed on the basis of the drug’s tolerability profile, the side effects identified during clinical trials, and the pharmacovigilance data (the French pharmacovigilance system monitors ODs available on the market). The OD efficacy assessment was specific to each drug, depending on the disease and target population. If an OD was better tolerated than its alternatives, despite lower efficacy, it would receive an SMR of “important” for a serious disease. In the next steps, this might exceptionally lead to reimbursement. The assessment of the efficacy profile of the OD had to confirm a sufficient level of evidence (or less robust evidence for innovative study designs) in the clinical context, based on an appropriate study design in terms of population, comparator, outcome measure, duration, inclusion and exclusion criteria, and statistical analysis. In France, in addition to the general safety and efficacy assessment, five main criteria (and related aspects) in the SMR influenced reimbursement decisions. The CT served as an advisory body for drug reimbursement. Once it has issued a positive recommendation for reimbursement (an SMR of “sufficient”), the National Union of Sickness Insurance Funds (in French, Union Nationale des Caisses D’assurance Maladie) set the reimbursement rate according to SMR assessment.

#### 3.1.5 Germany

In Germany, the safety and efficacy assessments of ODs were partly mandatory and had an informative function in the reimbursement process. Therefore, their impact could be described as “low.” There was a specific policy for ODs described in Social Code Book V (in German: Sozialgesetzbuch V, [SGB V]). This policy determined that ODs underwent HTA, but the result was only informative and did not influence reimbursement decisions as long as annual sales (measured by turnover), were below a certain threshold – 30 million Euro threshold since the beginning of 2023, before that, the threshold used to be higher (50 million Euro) - if an OD turns out to be above this threshold, it loses its special status, and comparative evidence will be required. Orphan drugs approved by the EMA were considered safe and effective by definition, but HTA information could be used in price negotiations. Thus, ODs had to show an acceptable safety and efficacy profile to support the authorization decision (and therefore reimbursement), and the procedure was the same for pediatric and adult ODs. Any clinical aspect that might affect the safety of an OD could be used to inform reimbursement decisions. Meanwhile, the methodology developed by the Institute for Quality and Efficiency in Healthcare (in German, Institut für Qualität und Wirtschaftlichkeit im Gesundheitswesen) was used to assess the clinical efficacy of the drug. In addition to general safety and efficacy assessment, other clinical aspects influencing reimbursement decisions included a comparison with the current standard of care (drug or other). However, by definition, this did not apply to ODs. An advisory body for drug reimbursement negotiations in Germany was the Federal Joint Committee (in German, Gemeinsamer Bundesausschuss, [G-BA]). Negotiations were conducted between the umbrella organization of the healthcare funds (GKV-Spitzenverband) and the individual manufacturer. For ODs, G-BA may require that there will have to be continuous data collection and analysis on the application of this drug, with the aim of generating “real world data” that may subsequently be used for assessing the benefit of the drug (article 130b in SGB V). Any evidence generated from this may subsequently be used to adapt reimbursed prices. Due to informative value of the HTA for ODs, no specific analyses were included in the HTA process. Consequently, the selection could be negotiated, allowing for the utilization of any analysis.

#### 3.1.6 Greece

Greece has implemented specific legislation to support the circulation and reimbursement of ODs, in line with the European Policy being implemented. Thus, according to paragraph 3, article 87 of Law 4472/17 (Official Government Gazette Α′ 74/19.05.2017), unlike non-orphan drugs, ODs were exempt from the obligation to be reimbursed in at least two-thirds of the EU Member States where they were marketed (not less than nine), and at least half of these Member States were specifically mentioned as having an HTA mechanism for medicines for human use. Moreover, it was not necessary for ODs to be priced (on a specific pharmaceutical form, content, and packaging) in at least three Eurozone Member States in order to be priced for the first time. Orphan drugs were priced even if prices were available in only two Eurozone Member States.

In Greece there were individual cases where a drug can be administered and reimbursed even if its efficacy profile has not yet been officially assessed. In the first scenario, the physician could request the approval of a drug (i.e., a drug that has not been assessed in Greece) for a specific patient and receive reimbursement after a positive opinion by the National Organization for the Provision of Healthcare Services (in Greek, Εθνικός Οργανισμός Παροχής Υπηρεσιών Υγείας). In the second scenario, if there was a need for an unapproved drug that was being evaluated by the EMA and was in an early access program, the physician could contact the corresponding Early Access Committee and obtain approval. In the third scenario, when a medicine was not approved for a specific indication, the physician could send a request to the Off-label Committee and obtain approval for a particular patient. In the efficacy assessment of the OD, numerous important aspects were considered, such as the type of the trial (randomized controlled trials, retrospective study, etc.), the phase of the trial (phase II, III, IV trials), and characteristics of the trial (control group, blinding, randomization, etc.). In Greece, in addition to the general assessment of safety and efficacy, other clinical aspects influencing reimbursement decisions included QoL, severity and burden of disease, mortality, results on OD tolerability, reliability of clinical trial data, clinical characteristics of patients, treatment line, and population size. In Greece, the Committee on Health Technology Assessment and Reimbursement of Medicinal Products for Human Use has been an advisory board for drug reimbursement since 2018 (Law 4512/Official Government Gazzete Α′5/17.01.2018). Additional analyses could be conducted during an HTA procedure, including the assessment of clinical benefit, effect on mortality/morbidity rates, safety and tolerability data, comparison with available reimbursed drug treatments, and degree of reliability of clinical trial data. The budget impact analysis included the cost-effectiveness ratio, the clinical characteristics of target patient population, the stage of treatment (the therapeutic algorithm) for which the drug is proposed for reimbursement, and the scope of the population that can receive this treatment.

#### 3.1.7 Iceland

In Iceland, the safety and efficacy assessment of ODs was not mandatory for the reimbursement process, so its impact could not be rated. Decision-makers used HTA results – including safety and efficacy evaluations – from other Nordic countries including Denmark, Norway, Sweden, and Finland. Iceland had no specific policy on ODs; however, there may have been specific legislation on the use and reimbursement of expensive new drugs (not necessarily ODs). All clinical aspects and components of the evaluation of ODs were dependent on the outcome of reviewed HTA analyses from other countries. In addition, there were situations where reimbursement was accepted for any registered indication (made by the EMA), but indications that were not accepted or not included in the reimbursement application were not reimbursed without scientific evidence. In Iceland, HTA for ODs was not mandatory, but there was an advisory body for drug reimbursement known as Drugs and Therapeutic Committees at university hospitals, which can also make reimbursement recommendations.

#### 3.1.8 Italy

In Italy, in the absence of therapeutic alternatives, patients with rare diseases could gain access to ODs through some legislative instruments that allow the use of a drug on a national basis (Law 648/1996), or through the possibility of individual prescription on a nominal basis (Law 326/2003 and Law 94/1998), or by regulating compassionate use (Ministerial Decree of 17 September 2017). In addition, orphan designation by the EMA provided additional benefits during national pricing and reimbursement negotiations.

Compassionate use allows for free access, with expenses borne by the pharmaceutical company, to experimental drugs, drugs authorized for different indications (i.e., off-label use) as well as drugs authorized but not yet available in the national territory. Following a positive opinion from the Committee for Medicinal Products for Human Use (CHMP) in EMA, manufacturers have the option to promptly submit their dossier, rather than waiting for the usual 3-month period after regulatory approval by the European Commission (2023). As such, they can receive priority in the process for pricing and reimbursement decisions by the Italian Medicines Agency [in Italian, Agenzia Italiana del Farmaco (AIFA)]. Finally, under certain conditions, AIFA might offer reimbursement for ODs before their regulatory approval. Additionally, as part of a dedicated program for independent research, the AIFA could provide funding for nonprofit research focused on ODs and rare diseases (Villa et al., 2022).

In Italy, the safety assessment of ODs was not strictly mandatory; therefore, there was no acceptable safety profile and no specific clinical aspects to be evaluated. There were also no separate guidelines for pediatric and adult patients (Marcellusi et al., 2023). The situation was slightly different for the acceptable efficacy profile. Efficacy assessment was mandatory in the reimbursement process, and the procedure was the same for pediatric and adult ODs. In particular, if pre-marketing data are lacking, the efficacy profile of OD could be evaluated based on: “results of already concluded phase II clinical trials” (Law 94/1998 and Law 648/1996) and/or “phase I clinical trials documenting the activity and safety of the medicinal product (excluding advanced therapies)” (Ministerial Decree of 17 September 2017). Thus, the phase of the clinical trial may have been one of the clinical aspects influencing reimbursement decisions. In addition, AIFA considered a new drug to be innovative – which was also important for ODs – if it met the following three criteria: 1) therapeutic need, 2) added therapeutic value, and 3) robustness of the scientific evidence submitted by the company together with the request for innovation (Xoxi et al., 2022). An advisory body for drug reimbursement in Italy was the National Center for Health Technology Assessment. Clinical analyses (e.g., general description of the disease, estimated number of patients who can be treated with the drug, description of medical needs, and description of the added therapeutic value and innovation) were required during the HTA. In addition, budget impact analyses and pharmacoeconomic evaluations were explicitly required for ODs.

#### 3.1.9 The Netherlands

In the Netherlands, safety and efficacy were assessed by two different government agencies. The safety assessment was required for market access and the efficacy assessment was one of the requirements for reimbursement. Netherlands had specific legislation and policy regarding ODs and therefore had a separate pathway for reimbursement decisions. This pathway was based on the “Policy for appraisal of orphan drugs” set by the Dutch Healthcare Institute (in Dutch, Zorginstituut Nederland), an independent administrative body that was a subsidiary of the Ministry of Health. Dutch legislation regarding medicines and reimbursement [the Medicines Law (in Dutch, Geneesmiddelenwet)] and Decision on Health Insurance [in Dutch, Besluit Zorgverzekering] simply state that it is the prerogative of the Dutch Healthcare Institute to advise the Ministry of Health on whether to reimburse medicines and/or to initiate measures to ensure proper use of them (for instance: cost effectiveness, data collection on long-term effects, etc.) These pieces of legislation do not differentiate between ODs and non-ODs but there are areas where differences have been applied. For ODs, the requirements for long-term efficacy data were less stringent than for non-ODs, but they were replaced by requirements for the collection of long-term efficacy data. However, the Dutch Healthcare Institute acknowledged that data on long-term effectiveness were often initially unavailable for ODs. In addition, there was a special reimbursement pathway for ODs known as “financial arrangements”, which aimed to increase their cost-effectiveness. ODs were subject to less stringent cost-effectiveness requirements, but only if they significantly improved the QoL and there were no other treatments for the disease. All newly submitted drugs were assessed by a pediatrics committee, except for medicines for conditions that did not affect children. In the assessment of the OD safety profile, all important aspects were addressed, including side effects, AEs, teratogenicity, interactions, and allergies. Orphan drugs had to show an acceptable efficacy profile to be reimbursed, and this acceptable profile was defined as a drug that should have the same level of efficacy as required for all other drugs or treatments, and ODs, if applicable, had to be at least as effective as the currently recommended treatment for the disease. In addition, a distinction was made between short-term and long-term efficacy. As data on long-term efficacy were often scarce, evaluations often required follow-up studies during the first years a medicine was on the market. Long-term outcomes were more valuable than short-term outcomes because they were less common for ODs. In the assessment of the efficacy profile of ODs, evidence was evaluated using the Grading of Recommendations, Assessment, Development, and Evaluations (GRADE) framework (Siemieniuk and Guyatt, 2024). In addition to the general safety and efficacy assessment, other clinical aspects influencing reimbursement decisions included the burden of the disease and cost (for the patient to pay), practicality (the possibility of carrying out the treatment in practice), and cost-effectiveness (acceptability of the cost in relation to the benefits of the treatment). In special circumstances, ODs can be reimbursed at the special request of a physician and are only available through parallel import. An advisory body for drug reimbursement in the Netherlands was the Dutch Healthcare Institute. The reimbursement of a drug with a negative recommendation was possible if there were no other treatments for the condition, if there was a sufficient improvement in QoL, or if there was sufficient certainty that careful patient selection would increase cost-effectiveness.

#### 3.1.10 Poland

In Poland, there was no specific legislation on ODs, but there were some policy mechanisms in place such as the National Plan for Rare Diseases (in Polish, Narodowy Plan dla Chorób Rzadkich). The plan was implemented in 2021 to improve access to diagnosis and treatment. Examples of activities include the Rare Disease Patient Passport and the Polish Rare Disease Registry. No specific criteria were used to assess the safety and efficacy profile of ODs, and the same aspects were considered as for non-ODs (frequency and severity of serious AEs, control group, blinding, randomization, treatment arms, etc.). In addition to the general assessment of safety and efficacy, other clinical aspects influencing reimbursement decisions included QoL, quality-adjusted life years, innovation of the new therapy, and lack of an alternative method of treatment (breakthrough therapy). Orphan drugs were usually reimbursed under the “drug program procedure” (also available for non-ODs) for a highly innovative drug or a drug with high clinical value. An advisory body for drug reimbursement in Poland was the Agency for Health Technology Assessment and Tariff System (in Polish, Agencja Oceny Technologii Medycznych i Taryfikacji). In addition to standard HTA analyses, decision problem analysis was also required.

#### 3.1.11 Scotland

Scotland had specific policy only for ultra-ODs (conditions with prevalence of 1 in 50,000 or less in Scotland, and requires specialist management). This policy was called the “Ultra-orphan pathway” and was a part of the “Rare Disease Action Plan” (the Scottish Government’s strategy) in addition, “Rare Disease Action Plan” supports the shared priorities of the 2021 United Kingdom Rare Diseases Framework. “Ultra-orphan pathway” developed by Scottish Medicines Consortium (SMC) started in 2018 and granted access for ultra-ODs for 3 years (on a case-by-case basis) before a decision was made on routine use to allow for the collection of further evidence – drugs need to have a United Kingdom orphan marketing authorization from Medicines and Healthcare products Regulatory Agency (MHRA). For ODs (or end of life drugs), manufacturers have the option to request a Patient and Clinical Engagement (PACE) meeting following a “not recommended” decision using the standard path. PACE meetings can be used “to describe the added benefits of the medicine, from both patient and clinician perspectives, that may not be fully captured within the conventional clinical and economic assessment process.” Approved drugs (by the MHRA, previously also EMA) with positive decisions by the relevant HTA authority (the National Institute for Health and Care Excellence, NICE in England or SMC in Scotland) can be prescribed, and will be fully paid for by the National Health Service (NHS). The safety assessment for ODs was mandatory but was not assessed by national institutions because the analysis performed by the EMA during the authorization process was considered sufficient. The evaluation of the safety profile of ODs in the reimbursement process focused on clinical and cost-effectiveness data, while risk assessment and marketing authorization were carried out by the EMA/MHRA. Orphan drugs had to demonstrate an acceptable efficacy profile in order to be reimbursed, with the exception of early access for ultra-ODs (as mentioned above). The acceptable efficacy profile was defined as cost-effectiveness rather than clinical benefit, but this was not a formal definition. As evidence could be limited, during the evaluation of the efficacy profile of ODs, all types of study designs were accepted in assessing the efficacy profile of ODs, including expert opinion if data were lacking. In addition to the general assessment of safety and efficacy, other clinical aspects influencing reimbursement decisions included QoL, cost-effectiveness, higher threshold values applied, higher levels of uncertainty accepted, often a wider social care cost, and the perspective of carers. The threshold is challenging as SMC don't have an explicit one and there is flexibility as they can apply modifiers. SMC never use an explicit £30K threshold (threshold more of a guide). Orphan and end of life drugs have to fit specific criteria – if they meet these criteria and ICER is >£30K the submission can go to PACE (patient and carer group input), and essentially “other” factors are considered, and it may get accepted above £30K ICER. No specific hard threshold or modifiers though (as they do at NICE).

#### 3.1.12 Spain

In Spain, the reimbursement assessment process was the same for ODs and non-ODs. Efficacy and safety data played an important role in reimbursement decisions and similar cost aspects were considered for both standard and orphan drugs. For safety assessment, all standard aspects were addressed, such as general safety or frequent and serious AEs. In exceptional cases, the safety profile of a drug could justify excluding patients at high risk of toxicity from drug reimbursement. Specific safety concerns in children (as a vulnerable group) could also theoretically justify exclusion from reimbursement. As with safety, there were exceptions where the efficacy of an OD was controversial (in terms of limited data or low clinical relevance of available results) at the time of evaluation, but the OD still received reimbursement. In the efficacy assessment of ODs, numerous standard aspects were addressed, such as study type and phase, control group, blinding, randomization, study duration, clinical relevance, and magnitude of the results.

Orphan drugs could be reimbursed under special conditions, for example, reimbursed in the authorized indication with a pharmaco-clinical protocol or reimbursed only for patients meeting specific clinical criteria. In Spain, there was no national HTA agency. The Spanish Agency of Medicines and Health Products (in Spanish, Agencia Española de Medicamentos y Productos Sanitarios [AEMPS]) coordinated the development of a document called a Therapeutic Positioning Report. This technical report included scientific information, clinical safety and efficacy assessment, safety, and rarely, economic information. However, it did not include recommendations for reimbursement.

Based on this information and some other factors (e.g., a price proposed by the MAH, number of potential patients) as well as after discussions with the MAH aimed at reaching an agreement, the Interministerial Commission on Drug Prices proposed a decision to the Ministry of Health on whether or not to finance the drug through the Spanish National Health System. Once the drug was approved by AEMPS, the MAH had to apply for its commercialization. Once the drug was assigned a national code, the General Directorate of Pharmacy of the Ministry of Public Health automatically initiated a financing study procedure, but the MAH had to submit the drug file and the economic proposal to continue the procedure. In addition to HTA assessments, epidemiological data on the disease were also assessed. The economic analysis was usually not publicly available.

### 3.2 The number of ODs with a positive recommendation or reimbursement in European countries

At the start of the study, as of September 2022, there were 136 drugs with orphan designation and authorized in the EMA web-based registry (Supplementary Appendix SA1). Orphan drugs were grouped according to ATC codes as follows:

– ATC A (Alimentary tract and metabolism; n = 28);
– ATC B (Blood and blood forming organs; n = 10);
– ATC C (Cardiovascular system; n = 3);
– ATC D (Dermatologicals; n = 2);
– ATC H (Systemic hormonal preparations, excluding sex hormones and insulins; n = 6);
– ATC J (Antiinfectives for systemic use; n = 11);
– ATC L (Antineoplastic and immunomodulating agents; n = 49);
– ATC M (Musculo-skeletal system; n = 7);
– ATC N (Nervous system; n = 9);
– ATC P (Antiparasitic products, insecticides and repellents; n = 1);
– ATC R (Respiratory system; n = 2);
– ATC S (Sensory organs; n = 5);
– ATC V (Various; n = 3).

The included countries differed in the number of ODs with a positive recommendation of advisory body for drug reimbursement and the number of reimbursed ODs (*p* < 0.001). The highest number of ODs with a positive recommendation was noted in Italy (n = 115, 84.6%) and the lowest in Iceland (n = 27, 19.9%). On the other hand, the highest number of reimbursed ODs was reported for Germany (n = 117, 86%) and the lowest for Iceland (n = 32, 23.5%) (*p* < 0.001). There were also significant differences between ODs with the same ATC code (Table 3); ODs based on ATC classifications with codes A, B, J, L and S were statistically significantly different between countries in terms of positive recommendation, while drugs with codes A, B, L and N were statistically significantly different between countries in terms of reimbursement.

**TABLE 3:** The frequency of ODs having a positive recommendation (in at least one of the indications) and those being reimbursed (in at least one of the indications) by country and ATC classification.

Anatomical therapeutic chemical (ATC) classification	Belgium	Finland	France	Germany	Greece	Iceland	Italy	Netherlands	Poland	Scotland	Spain	Mean %	*p*-value
Positive recommendations													
All ODs (N = 136)	50 (36.8%)	41 (30.2%)	102 (75%)	n/a	57 (41.9%)	27 (19.9%)	115 (84.6%)	90 (66.2%)	35 (25.7%)	58 (42.7%)	70 (51.5%)	(47.4%)	<0.001
A (N = 28)	6 (21.4%)	7 (25%)	19 (67.9%)	n/a	7 (25%)	1 (3.6%)	24 (85.7%)	17 (60.7%)	7 (25%)	9 (32.1%)	13 (46.4%)	(39.3%)	**<0.001**
B (N = 10)	4 (40%)	2 (20%)	7 (70%)	n/a	4 (40%)	2 (20%)	8 (80%)	7 (70%)	0 (0%)	4 (40%)	5 (50%)	(43%)	**0.005**
C (N = 3)	1 (33.3%)	1 (33.3%)	3 (100%)	n/a	1 (33.3%)	2 (66.7%)	1 (33.3%)	2 (66.7%)	1 (33.3%)	3 (100%)	2 (66.7%)	(56.7%)	0.506
D (N = 2)	0 (0%)	0 (0%)	1 (50%)	n/a	1 (50%)	0 (0%)	2 (100%)	1 (50%)	0 (0%)	0 (0%)	0 (0%)	(25%)	0.213
H (N = 6)	0 (0%)	2 (33.3%)	4 (66.7%)	n/a	4 (66.7%)	1 (16.7%)	4 (66.7%)	4 (66.7%)	1 (16.7%)	2 (33.3%)	4 (66.7%)	(43.3%)	0.085
J (N = 11)	3 (27.3%)	4 (36.4%)	7 (63.6%)	n/a	4 (36.4%)	1 (9.1%)	10 (90.9%)	6 (54.5%)	1 (9.1%)	4 (36.4%)	4 (36.4%)	(40%)	**0.003**
L (N = 49)	24 (49%)	15 (30.6%)	38 (77.6%)	n/a	28 (57.1%)	14 (28.6%)	43 (87.8%)	34 (69.4%)	19 (38.8%)	26 (53.1%)	25 (51%)	(54.3%)	**<0.001**
M (N = 7)	3 (42.9%)	4 (57.1%)	7 (100%)	n/a	2 (28.6%)	2 (28.6%)	6 (85.7%)	6 (85.7%)	2 (28.57%)	3 (42.9%)	4 (57.1%)	(55.7%)	0.036
N (N = 9)	4 (44.4%)	4 (44.4%)	8 (88.9%)	n/a	4 (44.4%)	2 (22.2%)	7 (77.8%)	6 (66.7%)	2 (22.2%)	4 (44.4%)	5 (55.6%)	(51.1%)	0.083
P (N = 1)	0 (0%)	0 (0%)	0 (0%)	n/a	0 (0%)	0 (0%)	1 (100%)	0 (0%)	0 (0%)	0 (0%)	0 (0%)	(10%)	0.350
R (N = 2)	2 (100%)	2 (100%)	2 (100%)	n/a	1 (50%)	0 (0%)	2 (100%)	2 (100%)	1 (50%)	0 (0%)	2 (100%)	(70%)	0.085
S (N = 5)	3 (60%)	0 (0%)	4 (80%)	n/a	1 (20%)	1 (20%)	5 (100%)	3 (60%)	1 (20%)	2 (40%)	4 (80%)	(48%)	**0.021**
V (N = 3)	0 (0%)	0 (0%)	2 (66.7%)	n/a	0 (0%)	1 (33.3%)	2 (66.7%)	2 (66.7%)	0 (0%)	1 (33.3%)	2 (66.7%)	(33.3%)	0.213
Reimbursement													
All ODs (N = 136)	50 (36.8%)	71 (52.2%)	83 (61%)	117 (86%)	60 (44.1%)	32 (23.5%)	103 (75.7%)	88 (64.7%)	50 (36.76%)	58 (42.7%)	73 (53.7%)	(52.5%)	**<0.001**
A (N = 28)	6 (21.4%)	11 (39.3%)	12 (42.9%)	24 (85.7%)	8 (28.6%)	3 (10.7%)	23 (82.1%)	16 (57.1%)	7 (25%)	9 (32.1%)	14 (50%)	(43.2%)	**<0.001**
B (N = 10)	4 (40%)	5 (50%)	7 (70%)	10 (100%)	4 (40%)	2 (20%)	7 (70%)	7 (70%)	4 (40%)	4 (40%)	6 (60%)	(54.6%)	**0.033**
C (N = 3)	1 (33.3%)	1 (33.3%)	2 (66.7%)	2 (66.7%)	1 (33.3%)	2 (66.7%)	1 (33.3%)	2 (66.7%)	2 (66.7%)	3 (100%)	2 (66.7%)	(57.6%)	0.839
D (N = 2)	0 (0%)	1 (50%)	1 (50%)	2 (100%)	1 (50%)	0 (0%)	1 (50%)	1 (50%)	0 (0%)	0 (0%)	0 (0%)	(31.8%)	0.400
H (N = 6)	0 (0%)	2 (33.3%)	4 (66.7%)	4 (66.7%)	4 (66.7%)	1 (16.7%)	2 (33.3%)	4 (66.7%)	1 (16.7%)	2 (33.3%)	3 (50%)	(40.9%)	0.160
J (N = 11)	3 (27.3%)	4 (36.4%)	6 (54.5%)	9 (81.8%)	4 (36.4%)	2 (18.2%)	8 (72.7%)	5 (45.5%)	3 (27.3%)	4 (36.4%)	4 (36.4%)	(43%)	0.072
L (N = 49)	24 (49%)	32 (65.3%)	31 (63.3%)	47 (95.9%)	29 (59.2%)	15 (30.6%)	40 (81.6%)	35 (71.4%)	25 (51%)	26 (53.1%)	29 (59.2%)	(61.8%)	**<0.001**
M (N = 7)	3 (42.9%)	4 (57.1%)	6 (85.7%)	5 (71.4%)	2 (28.6%)	2 (28.6%)	6 (85.7%)	5 (71.4%)	4 (57.1%)	3 (42.9%)	5 (71.4%)	(58.4%)	0.266
N (N = 9)	4 (44.4%)	5 (55.6%)	7 (77.8%)	8 (88.9%)	5 (55.6%)	2 (22.2%)	7 (77.8%)	5 (55.6%)	1 (11.1%)	4 (44.4%)	5 (55.6%)	(53.5%)	**0.034**
P (N = 1)	0 (0%)	0 (0%)	0 (0%)	0 (0%)	0 (0%)	0 (0%)	0 (0%)	1 (100%)	0 (0%)	0 (0%)	0 (0%)	(9.1%)	0.358
R (N = 2)	2 (100%)	2 (100%)	2 (100%)	2 (100%)	1 (50%)	1 (50%)	2 (100%)	2 (100%)	1 (50%)	0 (0%)	2 (100%)	(77.3%)	0.199
S (N = 5)	3 (60%)	2 (40%)	3 (60%)	2 (40%)	1 (20%)	1 (20%)	4 (80%)	3 (60%)	1 (20%)	2 (40%)	1 (20%)	(41.8%)	0.535
V (N = 3)	0 (0%)	2 (66.7%)	2 (66.7%)	2 (66.7%)	0 (0%)	1 (33.3%)	2 (66.7%)	2 (66.7%)	1 (33.3%)	1 (33.3%)	2 (66.7%)	(45.5%)	0.551

ATC, Anatomical Therapeutic Chemical |n/a - not applicable (country-level HTA, recommendation is not required for ODs, in Germany) ! Data from Austria was missing, because covered only outpatient sector.

Bold values mean than p-values are less than 0.05.

Five drugs (migalastat, pomalidomide, sorafenib, macitentan, and midostaurin) had the highest number of positive recommendations among the countries analyzed (n = 10; Supplementary Appendix SA2) – three of them had ATC code L. The most frequently reimbursed drug in all countries was macitentan (n = 12; Supplementary Appendix SA3) – ATC code C.

### 3.3 Correlations of clinical aspects with OD reimbursement and HTA recommendations

Impact of safety and efficacy assessment on reimbursement ODs decisions; dedicated to ODs legislation and policies; safety assessment; efficacy assessment; rule “reimbursement decisions dependent on EMA authorization status and ODs designation”; special advisory institution that makes recommendations; obligatory HTA; rule “positive recommendation (from HTA agency/advisory body) ensures reimbursement”; rule “negative recommendation excludes reimbursements” and ICER/ICUR thresholds the same for ODs as for non-ODs were not statistically significantly correlated with positive recommendation and reimbursement (Table 4).

**TABLE 4:** Univariate associations between the country policy for ODs; ATC classification and positive recommendation or reimbursement of an ODs.

Characteristics		Positive recommendation		Reimbursement	
		OR (95% CI)	*p*-value	OR (95% CI)	*p*-value
Anatomical Therapeutic Chemical (ATC) classification	ATC B vs. ATC A	1.20 (0.72–2.01)	0.474	2.09 (0.86–5.11)	0.104
	ATC C vs. ATC A	2.34 (1.02–5.36)	**0.046**	2.47 (0.69–8.75)	0.163
	ATC D vs. ATC A	0.45 (0.14–1.40)	0.167	0.45 (0.10–2.08)	0.306
	ATC H vs. ATC A	1.22 (0.66–2.28)	0.522	0.85 (0.38–1.93)	0.706
	ATC J vs. ATC A	1.04 (0.63–1.70)	0.886	0.99 (0.52–1.87)	0.977
	ATC L vs. ATC A	2.08 (1.50–2.89)	**<0.001**	3.35 (1.16–9.69)	**0.026**
	ATC M vs. ATC A	2.23 (1.25–3.98)	**0.007**	2.66 (0.90–7.83)	0.076
	ATC N vs. ATC A	1.79 (1.06–3.02)	**0.030**	1.95 (0.82–4.65)	0.132
	ATC P vs. ATC A	0.13 (0.01–1.13)	0.065	0.05 (0.00–1.61)	0.090
	ATC R vs. ATC A	4.59 (1.59–13.24)	**0.005**	10.88 (0.95–124.67)	0.055
	ATC S vs. ATC A	1.54 (0.79–2.99)	0.204	0.91 (0.38–2.17)	0.836
	ATC V vs. ATC A	0.73 (0.30–1.76)	0.484	1.15 (0.39–3.42)	0.796
Impact of safety and efficacy assessment on reimbursement orphan drugs (ODs) decisions	[high vs. other]	4.29 (0.77–23.89)	0.097	0.81 (0.22–3.02)	0.749
Dedicated to ODs legislation and policies	[present vs. absent]	0.46 (0.16–1.36)	0.160	0.95 (0.29–3.13)	0.938
Safety assessment	[mandatory vs. voluntary/none]	0.74 (0.17–3.12)	0.681	1.21 (0.26–5.67)	0.806
Efficacy assessment	[mandatory vs. voluntary/none]	4.29 (0.77–23.89)	0.097	5.29 (0.01–4,446.92)	0.628
Reimbursement decisions dependent on European Medicines Agency authorization status and ODs designation	[yes vs. no]	0.44 (0.16–1.26)	0.128	0.60 (0.12–2.98)	0.534
Special advisory institution that makes recommendations	[yes vs. no]	1.45 (0.35–6.03)	0.611	1.01 (0.26–3.92)	0.989
Obligatory HTA	[yes vs. no]	2.99 (0.83–10.74)	0.094	4.53 (0.19–105.30)	0.347
Positive recommendation (from HTA agency/advisory body) ensures reimbursement	[yes vs. no]	0.64 (0.20–2.01)	0.441	0.58 (0.11–3.20)	0.532
Negative recommendation (from HTA agency/advisory body) excludes reimbursement	[yes vs. no/partially]	0.23 (0.04–1.30)	0.097	0.19 (0.00–153.62)	0.626
ICER/ICUR thresholds the same for ODs as for non-ODs	[yes vs. no]	1.16 (0.37–3.69)	0.797	1.05 (0.33–3.29)	0.938

HTA, Health Technology Assessment | ICER, Incremental Cost-Effectiveness Ratio | ICUR, Incremental Cost Utility Ratio | ATC, Anatomical Therapeutic Chemical classification | CI, Confidence Interval | OR, Odds Ratio ! All models had significant (*p* < 0.05), non-zero between-country variance and non-zero between-drug variance, which suggests occurrence of other aspects, not measured in this study, that are correlated with positive recommendation or reimbursement of OD.

Bold values mean than p-values are less than 0.05.

However, significant correlations were found for ATC codes. ODs with ATC codes C (cardiovascular system), L (antineoplastic and immunomodulating agents), M (musculo-skeletal), N (nervous system) and R (respiratory system) were more likely to receive a positive recommendation or reimbursement compared with ODs with other codes (Table 4).

## 4 Discussion

This study investigated the reimbursement policy for ODs in selected European countries in relation to the availability and impact of clinical evidence, selected aspects (additional clinical determinants, regulatory context, EMA authorization status) and HTA procedures. Furthermore, the study identified the drug type according to ATC and other factors that contribute to the discrepancy between the number of drugs with a positive recommendation and those that have received reimbursement.

Our results showed that, with some exceptions, the safety and efficacy assessment was mandatory in the OD policy and was rated the highest on the three-point scale. Additionally, an acceptable safety profile for ODs was rarely defined (in one country), and it was more common to define an acceptable efficacy profile (in four countries). With rare exceptions, the acceptable safety and efficacy profiles of ODs were similar for children and adults.

We examined reimbursement policies in selected European countries to identify factors responsible for the differences in the number of medicines with a positive recommendation *versus* the number of medicines that obtained reimbursement. The results showed heterogeneity in the rates of positive recommendation (12.5%–84.6%) and reimbursement (12.5%–86%) for ODs in each country, which is consistent with the results of previous studies (Malinowski et al., 2018; Stawowczyk et al., 2019; Kawalec et al., 2016). In addition to three countries (Austria, Belgium, Sweden), there were drugs that were reimbursed even though they were not on the list of recommended drugs. Our results are consistent with those reported by Kawalec et al. (Kawalec et al., 2016), who showed that 5.4% of ODs that were not assessed by any of the eight European HTA agencies (did not receive a reimbursement recommendation) still received reimbursement. The authors reviewed 101 ODs authorized by the EMA between 2002 and 2015. This implies that the reimbursement status does not always correspond to the type of recommendation made by an HTA agency (or an advisory body) for an OD.

In six countries analyzed in our study, there was specific legislation or policy regulating access to ODs, but this was not associated with an increase in the number of reimbursed drugs. However, the situation has changed slightly because in a study by Sarnola et al. (2018) only two of the 24 European countries surveyed had specific policies in place for assessing the reimbursement status. Nevertheless, it should be noted that our study used a slightly broader definition of a drug policy. The investigators suggested that the reimbursement policy for ODs was usually the same as for other drugs. According to another study (Kawalec et al., 2016), the reimbursement rates for ODs were higher (as a percentage of all evaluated ODs) in countries that had specific criteria for ODs, such as Germany, the Netherlands, and Sweden. Therefore, the availability of additional requirements for ODs in the HTA and reimbursement processes may influence patient access to such medicines (Kawalec et al., 2016). One of the components of a drug policy is budget planning and expenditure on drugs. Although this was not the focus of our study, it should be mentioned that differences in the number of reimbursed drugs may also be influenced by national budgets for medicines (Malinowski et al., 2018; Sarnola et al., 2018; Picavet et al., 2012; Zelei et al., 2016). According to our respondents, selected country policies (e.g., clinical aspects) were an important part of the evaluation of ODs. However, the results of our statistical analysis did not show that they had a significant impact on the positive recommendation or reimbursement of ODs.

Our study showed that the HTA (together with various economic or clinical analyses) played an important role in the reimbursement process for ODs. The only countries where it was not mandatory were Belgium and Iceland, while in Germany it had only an informative function. In addition, most countries (except for Finland and Spain) had specific advisory bodies that provided opinions on ODs. Decision-makers are increasingly adapting their reimbursement procedures to consider the unique characteristics of ODs (Nicod et al., 2019). This has led to the development of a wide range of value assessment methodologies for ODs, known as value assessment frameworks (VAFs). Using these adapted frameworks, decision-makers attempt to strike a balance between traditional efficiency criteria, such as cost-effectiveness, and less common criteria, such as disease severity and unmet need (Blonda et al., 2021). Value assessment frameworks can include no or standard economic evaluation, variable incremental cost-effectiveness ratio threshold, weighted quality-adjusted life years, multi-criteria decision analysis, and a separate VAF (different from the standard VAF) (Blonda et al., 2021).

In our study, experts indicated that the HTA dossier could include different analyses. This is in line with a study by Brenna et al. (2020), which also showed that the implementation of an HTA dossier was not homogeneous. This was partially explained by Blonda et al. (2022), who suggested that the lack of data, such as insufficient evidence on the efficacy of ODs and the lack of local data on costs and utilities, often makes it difficult to estimate the value of ODs. In addition, while most ODs do not meet current cost-effectiveness thresholds, policymakers often concluded that patients with rare diseases need access to therapies regardless of cost-effectiveness (Blonda et al., 2022; Michel and Toumi, 2012) It was also argued that ODs should not be evaluated using standard approaches (increasing the importance of disease severity, the lack of adequate alternative treatments, ethical principles, and social solidarity) (Sarnola et al., 2018; Michel and Toumi, 2012). As a result, the EU regulation (Regulation [EU] 2021/2282 of the European Parliament and of the Council of 15 December 2021 on health technology assessment and amending Directive 2011/24/EU) [known as, Health Technology Assessment Regulation; HTAR] was established with consequent changes in international HTA (Regulation). This regulation aims to facilitate access to innovative medical products such as medical devices and medicines for EU patients – products frequently introduced with insufficient evidence to expedite market access for patients (European Parliament et al., 2024). HTAR delineates guidelines for joint clinical assessments (JCAs) and the formation of a coordination committee for national or regional health technology assessment (HTA) authorities (European Parliament et al., 2024). As of 2028, the assessment of clinical aspects (safety, efficacy) within an HTA dossier for ODs will be performed at the international level, while non-clinical evaluations (economic, social, ethical, etc.) will be assigned to national agencies (Regulation on Health Technology Assessment, 2024). This regulation establishes a permanent framework for cooperation to replace the existing system based on a voluntary network of national authorities (HTA Network) and project-based cooperation funded by the EU (Joint Actions EUnetHTA) (Regulation; EUnetHTA, 2024).

Our study revealed that ODs with ATC code L (“antineoplastic and immunomodulating agents”) represented the largest group of reimbursed medicines. However, a drug that was reimbursed in all 12 countries was classified as ATC C (“cardiovascular system”) (INN: macitentan; indication, hypertension, and pulmonary diseases). Countries differed in the rates of recommended or reimbursed drugs, and ATC codes were significantly correlated with reimbursement statuses and positive recommendations.

In our previous study (Jakubowski et al., 2024), we analyzed OD reimbursement policies in selected CEE countries. The current study showed that the share of reimbursed ODs was significantly higher in the studied countries (including mostly Western European [WE] countries) than in the CEE countries. Both studies revealed differences in national reimbursement policies for ODs and both confirmed that the ATC classification has a significant impact on the chances of receiving a positive recommendation and reimbursement. In addition, the previous study showed that these chances were also significantly influenced by the safety and efficacy assessment as well as specific clinical aspects. Reimbursement policies for ODs in WE countries seem to be more harmonized and differences among countries were less pronounced than in CEE countries. This can be evidenced by the possibility of joint cross-country evaluations of ODs and the example of the BeNeLuxAI initiative (Vogler et al., 2021). This initiative launched in 2015 brings together Belgium, the Netherlands, Luxembourg, Austria and Ireland to look at OD issues from a lifecycle value chain perspective. The BeNeLuxAI has worked together on horizon scanning (method to identify early developments) and HTA in addition to shared price negotiations. As a result of these collaborations, terms and methods for health technology assessment have been harmonized (Vogler et al., 2021).

Some investigators suggested that the classification of ODs by indication also has an indirect impact on reimbursement. Malinowski et al. (2019) showed that disease type (oncological, metabolic, and other) significantly influenced reimbursement decisions in four of the 10 countries analyzed, with positive decisions for oncological diseases significantly outweighing those for other diseases. On the other hand, (Kawalec et al., 2016) used the categories of ultra-ODs (for very rare diseases), oncological ODs, non-ultra-ODs, and non-oncological ODs, and reported the highest reimbursement rate for ultra-ODs (25%), as compared with the rate of about 20%–21% for the other groups. Orphan drugs for the treatment of oncological diseases constituted a single common group of ODs with an impact on reimbursement that recurred in the above classifications.

To gain a broader perspective, we identified some relevant publications for comparison. In 2020-2021, Blonda et al. (Blonda et al., 2022) conducted a qualitative survey among 22 European specialists (from 19 different countries), followed by in-depth semi-structured interviews, to investigate how to optimize the value assessment and appraisal of ODs for reimbursement purposes. The study showed that different countries use different reimbursement procedures to provide access to ODs, which is in line with our findings. The study highlighted the importance of transparency and trust in OD reimbursement and the need for a clear framework for decision-making, while leaving room for continuous improvement. It also identified several contextual determinants that have a particular impact on the evaluation process, such as the influence of bias and a general lack of consideration of opportunity costs (of ODs vs. non-ODs). It was suggested that this could improve the level of the arguments throughout the HTA and in the subsequent reimbursement agreement. Our study showed that in seven countries (>50%) the registration status or designation by the EMA had a significant impact on reimbursement decisions. Interestingly, Malinowski et al. (Malinowski et al., 2018) revealed that the authorization status granted by the EMA may be directly related to the reimbursement of ODs. The authors reported that conditional approval significantly reduced the chance of reimbursement in France, Italy, and Spain (by 77%–80%). However, approval granted under exceptional circumstances had a significant impact in Germany, with an 85% reduction in the chance of reimbursement. Blonda et al. (2022) suggested that a link between EMA decisions and national policies can lead to implicit bias, as it becomes even more difficult for decision-makers to refuse reimbursement. Even if their decisions are based on clinical efficacy and safety assessment rather than on national comparative cost-effectiveness, the public may find it difficult to accept that a decision-maker would “overrule” a global organization like the EMA.

Czech et al. (2020) conducted a systematic review of OD reimbursement in 12 selected Eurasian countries with the aim of analyzing legislation and health policy for rare diseases. They argued that differences in national legislation, healthcare budgets, health insurance, and reimbursement systems lead to inequalities in patient access to novel ODs. According to the authors, there are significant differences in regional reimbursement policies, and there is a general trend towards stricter reimbursement guidelines for expensive ODs. Every country should have early access programs in place. Such programs are relatively easy to implement and can temporarily satisfy urgent medical needs for ODs at little financial cost to society or patients. Although exemptions and reduced data requirements are often available in some form, no specific HTA procedures for ODs were identified. Despite the lack of evidence, many countries continue to fund ODs using regular HTA procedures. Czech et al. (2020) concluded that none of the countries analyzed in the review could be considered as having the “optimal” treatments for rare diseases.

Chan et al. (2020) reviewed OD policies in 194 countries and six subject areas. One of the areas that corresponding to the scope of our study was “safety and efficacy requirements.” The authors identified 92 countries (46.0%) with laws, regulations, or policies that facilitate patient access to ODs. Europe was found to have the highest rate of established OD policies (42 of 54, 77.8%) and Africa the lowest (6 of 47, 12.8%). In non-high-income countries, the rate of OD policies gradually increased between 2013 and 2019. Safety and efficacy requirements in OD policies were reported in 44 of 92 countries (47.8%). Risk minimization plans, pharmacovigilance programs, and updated OD reports from countries with a developed pharmaceutical industry were accepted as sufficient evidence of safety. While the study by Chan et al. (2020) did not summarize efficacy, the authors noted that ODs may not be readily available due to the frequent lack of scientific evidence for new medicines. They concluded that even with the current framework for OD policy, inconsistencies in internal policy could have unexpected negative consequences for a country if the accessibility and affordability of ODs were not balanced.

Last but not least, Rare 2030 project, a 2-year foresight study, is also noteworthy (Kole and Hedley, 2021). Its goal was to collect the opinions of patients, medical professionals, and policymakers in order to suggest changes to the European rare disease policy. In February 2021, the project’s proposals were delivered to the European Parliament and the following recommendations (some already implemented) were highlighted: create a European policy framework for national plans; improve diagnosis with harmonized standards and new technologies; build a healthcare ecosystem for consistent care; integrate patients into society and the economy; foster participation; prioritize research; maximize data use, and improve treatment accessibility through investment and innovation (Kole and Hedley, 2021).

## 5 Strengths and limitations

Although our study was designed and conducted in a way to ensure maximum reliability, it has some limitations. First, not all European countries were included in the study, despite our intention to do so. The data collected from Austria on recommendations and reimbursement were limited because they covered only the outpatient sector. Moreover, a cross-comparison of OD policies should not be made without considering the underlying cultural, economic, health, and political issues, as each country’s health system operates in a specific local context. In addition, it was difficult to create a universal questionnaire due to differences in reimbursement procedures between countries. Therefore, it is likely that we did not obtain data on all policies adopted for ODs. We assessed drugs with an orphan designation granted in 2022, and OD policies as well as access to ODs in different countries may have changed since then. In addition, it was not possible to standardize the expenditure and copayment variables, which also had several shortcomings, so they were not used in the analysis. In some cases, the statistical coefficient might have been affected by the levels and frequencies of the variables analyzed. Therefore, the results of the statistical analysis should be interpreted with caution. Finally, we cannot exclude the possibility that other factors may have contributed to the observed results. Multivariate models were not tested because country and drug characteristics were often correlated with and/or dependent on one another.

Despite the above limitations, our study has several strengths. It provides up-to-date information on the reimbursement policies for ODs in up to 12 European countries. It also provides valuable information on the introduction, distribution, and reimbursement of recently approved ODs in Europe. We collected data from local national experts and, where necessary, asked for clarification to best explain the mechanisms of reimbursement procedures for ODs. The use of the ATC classification has provided a better understanding of the structure of the ODs themselves and the possible implications for reimbursement decisions. The aggregated summaries and country profiles can be useful for the development of a consistent international policy on ODs in the future.

## 6 Conclusion

Our study revealed differences between European countries in OD reimbursement policy in terms of the assessment of safety and efficacy (definition, evidence, populations), the level of impact of clinical assessment, additional clinical aspects, specific legislation and policies on ODs and EMA authorization decisions. It also showed that HTA was not mandatory in all countries (e.g., Belgium and Iceland) or only had an informative function (Germany), and that ODs could be reimbursed regardless of whether they received a positive or negative recommendation. The most common HTA analysis for ODs in the countries studied was cost-effectiveness analysis. Countries differed in the rates of recommended or reimbursed drugs and ATC codes were significantly correlated with reimbursement status and positive recommendation – the largest group was antineoplastic and immunomodulating agents (ATC L).

## Data Availability

The raw data supporting the conclusions of this article will be made available by the authors, without undue reservation.

## Abbreviations

AEs, Adverse events; ATC, Anatomical Therapeutic Chemical; CEE, Central Eastern European; EMA, European Medicines Agency; EU, European Union; HTA, Health technology assessment; MAH, Marketing authorization holder; n/a, Not available; OD, Orphan drug; QoL, Quality-of-life.

## References

[B1] Blonda A. Denier Y. Huys I. Kawalec P. Simoens S. (2022). How can we optimize the value assessment and appraisal of orphan drugs for reimbursement purposes? A qualitative interview study across European countries. Front. Pharmacol. 13, 902150. 10.3389/FPHAR.2022.902150 35928274 PMC9343828

[B2] Blonda A. Denier Y. Huys I. Simoens S. (2021). How to value orphan drugs? A review of European value assessment frameworks. Front. Pharmacol. 12, 631527. 10.3389/FPHAR.2021.631527 34054519 PMC8150002

[B3] Brenna E. Polistena B. Spandonaro F. (2020). The implementation of health technology assessment principles in public decisions concerning orphan drugs. Eur. J. Clin. Pharmacol. 76, 755–764. 10.1007/S00228-020-02855-7 32219539

[B4] Chan A. Y. L. Chan V. K. Y. Olsson S. Fan M. Jit M. Gong M. (2020). Access and unmet needs of orphan drugs in 194 countries and 6 areas: a global policy review with content analysis. Value Health 23, 1580–1591. 10.1016/J.JVAL.2020.06.020 33248513

[B5] Czech M. Baran-Kooiker A. Atikeler K. Demirtshyan M. Gaitova K. Holownia-Voloskova M. (2020). A review of rare disease policies and orphan drug reimbursement systems in 12 eurasian countries. Front. Public Health 7, 416. 10.3389/fpubh.2019.00416 32117845 PMC6997877

[B6] Detiček A. Locatelli I. Kos M. (2018). Patient access to medicines for rare diseases in European countries. Value Health 21, 553–560. 10.1016/j.jval.2018.01.007 29753352

[B7] Dupont A. G. Van Wilder P. B. (2011). Access to orphan drugs despite poor quality of clinical evidence. Br. J. Clin. Pharmacol. 71, 488–496. 10.1111/J.1365-2125.2010.03877.X 21395641 PMC3080635

[B8] EUnetHTA (2024). EUnetHTA. Available at: https://www.eunethta.eu/jointhtawork/(Accessed October 9, 2023).

[B9] European Commission (2023). Orphan medicinal products 2023. Available at: https://health.ec.europa.eu/medicinal-products/orphan-medicinal-products_en (Accessed October 8, 2023).

[B10] European Medicines Agency (2024a). Medicines. Available at: https://www.ema.europa.eu/en/medicines (Accessed May 16, 2022).

[B11] European Medicines Agency (2024b). Orphan designation: overview 2023. Available at: https://www.ema.europa.eu/en/human-regulatory/overview/orphan-designation-overview (Accessed October 8, 2023).

[B12] European Parliament. Kamphuis. B. De Jongh T. Bastiaanssen V. (2024). Tackling rare diseases – challenges, opportunities and gaps for action on rare diseases in the European Union.

[B13] Felippini A. Biglia L. V. Lima T. de M. Aguiar P. M. (2024). HTA criteria adopted in different models of public healthcare systems for orphan drugs: a scoping review. Health Policy (New York) 144, 105080. 10.1016/J.HEALTHPOL.2024.105080 38733643

[B15] Howie L. J. Hirsch B. R. Abernethy A. P. (2013). A comparison of FDA and EMA drug approval: implications for drug development and cost of care. Oncology 27, 1195–1200. 24624536

[B16] Jakubowski S. Kawalec P. Holko P. Kowalska-Bobko I. Kamusheva M. Petrova G. (2024). Clinical aspects of reimbursement policies for orphan drugs in Central and Eastern European countries. Front. Pharmacol. 15, 1369178. 10.3389/FPHAR.2024.1369178 38523639 PMC10957562

[B17] Kawalec P. Sagan A. Pilc A. (2016). The correlation between HTA recommendations and reimbursement status of orphan drugs in Europe. Orphanet J. Rare Dis. 11, 122. 10.1186/S13023-016-0501-4 27600717 PMC5012088

[B18] Kole A. Hedley V. (2021). Recommendations from the Rare 2030 Foresight Study: the future of rare diseases starts today.

[B19] Malinowski K. P. Kawalec P. Trabka W. Czech M. Petrova G. Manova M. (2019). Reimbursement legislations and decision making for orphan drugs in central and eastern european countries. Front. Pharmacol. 10, 487. 10.3389/FPHAR.2019.00487 31139080 PMC6518361

[B20] Malinowski K. P. Kawalec P. Trabka W. Sowada C. Pilc A. (2018). Reimbursement of orphan drugs in Europe in relation to the type of authorization by the European medicines agency and the decision making based on health technology assessment. Front. Pharmacol. 9, 1263. 10.3389/FPHAR.2018.01263 30483124 PMC6240661

[B21] Marcellusi A. Raimondo P. Galeone C. Canonico P. L. (2023). Time to market access in Italy: duration of the P&R process for rare disease drugs. Glob. and Regional Health Technol. Assess. 10, 79–88. 10.33393/GRHTA.2023.2610 PMC10628501 37942299

[B22] Marino M. Jamal Z. Siccardi M. A. (2023). Pharmaceutics. StatPearls. 30570996

[B23] Michel M. Toumi M. (2012). Access to orphan drugs in Europe: current and future issues. Expert Rev. Pharmacoecon Outcomes Res. 12, 23–29. 10.1586/ERP.11.95 22280193

[B24] Nicod E. Annemans L. Bucsics A. Lee A. Upadhyaya S. Facey K. (2019). HTA programme response to the challenges of dealing with orphan medicinal products: process evaluation in selected European countries. Health Policy (New York) 123, 140–151. 10.1016/J.HEALTHPOL.2017.03.009 28400128

[B25] Picavet E. Annemans L. Cleemput I. Cassiman D. Simoens S. (2012). Market uptake of orphan drugs – a European analysis. J. Clin. Pharm. Ther. 37, 664–667. 10.1111/J.1365-2710.2012.01364.X 22731105

[B26] Regulation on health technology assessment (2024). Available at: https://health.ec.europa.eu/health-technology-assessment/implementation-regulation-health-technology-assessment_en (Accessed October 9, 2023).

[B27] Regulation (EU). 2021/2282 of the European Parliament and of the Council of 15 December 2021 on health technology assessment and amending Directive 2011/24/EU.

[B28] Sarnola K. Ahonen R. Martikainen J. E. Timonen J. (2018). Policies and availability of orphan medicines in outpatient care in 24 European countries. Eur. J. Clin. Pharmacol. 74, 895–902. 10.1007/S00228-018-2457-X 29632962

[B14] Siemieniuk R. Guyatt G. (2024). What is GRADE? BMJ Best. Pract. Available at: https://bestpractice.bmj.com/info/toolkit/learn-ebm/what-is-grade/ (Accessed October 9, 2023).

[B29] Stawowczyk E. Malinowski K. P. Kawalec P. Bobiński R. Siwiec J. Panteli D. (2019). Reimbursement status and recommendations related to orphan drugs in European countries. Front. Pharmacol. 10, 1279. 10.3389/FPHAR.2019.01279 31827433 PMC6890830

[B30] U.S. Food and Drug Administration (2024). Orphan drug Act - relevant excerpts 2018. Available at: https://www.fda.gov/industry/designating-orphan-product-drugs-and-biological-products/orphan-drug-act-relevant-excerpts (Accessed October 8, 2023).

[B31] Villa F. Di Filippo A. Pierantozzi A. Genazzani A. Addis A. Trifirò G. (2022). Orphan drug prices and epidemiology of rare diseases: a cross-sectional study in Italy in the years 2014–2019. Front. Med. (Lausanne) 9, 820757. 10.3389/FMED.2022.820757 35252257 PMC8891228

[B32] Vogler S. Haasis M. A. van den Ham R. Humbert T. Garner S. Suleman F. (2021). European collaborations on medicine and vaccine procurement. Bull. World Health Organ 99, 715–721. 10.2471/BLT.21.285761 34621089 PMC8477421

[B33] Xoxi E. Di Bidino R. Leone S. Aiello A. Prada M. (2022). Value assessment of medicinal products by the Italian medicines agency (AIFA) and French national authority for health (HAS): similarities and discrepancies. Front. Med. Technol. 4, 917151. 10.3389/FMEDT.2022.917151 36134249 PMC9483157

[B34] Zelei T. Molnár M. J. Szegedi M. Kaló Z. (2016). Systematic review on the evaluation criteria of orphan medicines in Central and Eastern European countries. Orphanet J. Rare Dis. 11, 72–11. 10.1186/S13023-016-0455-6 27259284 PMC4893267

[B35] Zimmermann B. M. Eichinger J. Baumgartner M. R. (2021). A systematic review of moral reasons on orphan drug reimbursement. Orphanet J. Rare Dis. 16, 292. 10.1186/S13023-021-01925-Y 34193232 PMC8247078

