# Supplementary material for: Clinical and non-clinical aspects of reimbursement policy for orphan drugs in selected European countries
Source: Front Pharmacol. 2024 Nov 19;15:1498386. doi: 10.3389/fphar.2024.1498386 (PMC11611580; doi:10.3389/fphar.2024.1498386)
Supplement: Supplementary file 1 [file Table1.DOCX]

ONLINE ONLY SUPPLEMENTARY INFORMATION

###### Appendix 1 (Table) EMA-registered orphan drugs (valid for 12^th^ August 2022) – alphabetical order for ATC classification

| **No** | **ATC classification** | **International non-proprietary name (INN) or common name** | **Trade name** | **Therapeutic area (MeSH)** |
| --- | --- | --- | --- | --- |
| 1 | A05AA01 | Chenodeoxycholic acid | Chenodeoxycholic acid Leadiant | Xanthomatosis, Cerebrotendinous; Metabolism, Inborn Errors |
| 2 | A05AA03 | Cholic acid | Orphacol | Digestive System Diseases Metabolism, Inborn Errors |
| 3 | A05AA04 | Obeticholic acid | Ocaliva | Liver Cirrhosis, Biliary |
| 4 | A05AX | Odevixibat | Bylvay | Cholestasis, Intrahepatic |
| 5 | A07EA06 | Budesonide | Jorveza | Esophageal Diseases |
| 6 | A07EA06 | budesonide | Kinpeygo | Glomerulonephritis, IGA |
| 7 | A08AA | Setmelanotide | Imcivree | Obesity |
| 8 | A10BB01 | Glibenclamide | Amglidia | Diabetes Mellitus |
| 9 | A16 | Sebelipase alfa | Kanuma | Lipid Metabolism, Inborn Errors |
| 10 | A16A | telotristat ethyl | Xermelo | Carcinoid Tumor Neuroendocrine Tumors |
| 11 | A16AA | Metreleptin | Myalepta | Lipodystrophy, Familial Partial |
| 12 | A16AA04 | Mercaptamine | Procysbi | Cystinosis |
| 13 | A16AB | Cerliponase alfa | Brineura | Neuronal Ceroid-Lipofuscinoses |
| 14 | A16AB | Asfotase alfa | Strensiq | Hypophosphatasia |
| 15 | A16AB10 | Velaglucerase alfa | Vpriv | Gaucher Disease |
| 16 | A16AB12 | elosulfase alfa | Vimizim | Mucopolysaccharidosis IV |
| 17 | A16AB15 | Velmanase alfa | Lamzede | alpha-Mannosidosis |
| 18 | A16AB18 | Vestronidase alfa | Mepsevii | Mucopolysaccharidosis VII |
| 19 | A16AB19 | Pegvaliase | Palynziq | Phenylketonurias |
| 20 | A16AB25 | olipudase alfa | Xenpozyme | Acid sphingomyelinase deficiency (ASMD) type A/B or type B |
| 21 | A16AB26 | eladocagene exuparvovec | Upstaza | Amino Acid Metabolism, Inborn Errors |
| 22 | A16AX | Migalastat | Galafold | Fabry Disease |
| 23 | A16AX08 | Teduglutide | Revestive | Malabsorption Syndromes |
| 24 | A16AX09 | Glycerol phenylbutyrate | Ravicti | Urea Cycle Disorders, Inborn |
| 25 | A16AX10 | Eliglustat | Cerdelga | Gaucher Disease |
| 26 | A16AX16 | Givosiran | Givlaari | Porphyrias, Hepatic |
| 27 | A16AX18 | Lumasiran | Oxlumo | Hyperoxaluria, Primary |
| 28 | A16AX20 | lonafarnib | Zokinvy | Progeria Laminopathies |
| 29 | B01A | Caplacizumab | Cablivi | Purpura, Thrombotic Thrombocytopenic |
| 30 | B01AC21 | Treprostinil | Trepulmix | Hypertension, Pulmonary |
| 31 | B01AX01 | Defibrotide | Defitelio | Hepatic Veno-Occlusive Disease |
| 32 | B02BD04 | Eftrenonacog alfa | Alprolix | Hemophilia B |
| 33 | B02BD04 | Albutrepenonacog alfa | Idelvion | Hemophilia B |
| 34 | B02BD13 | Human coagulation factor X | Coagadex | Factor X Deficiency |
| 35 | B03XA06 | Luspatercept | Reblozyl | Anemia; Myelodysplastic Syndromes; beta-Thalassemia |
| 36 | B06AC05 | Lanadelumab | Takhzyro | Angioedemas, Hereditary |
| 37 | B06AX01 | Crizanlizumab | Adakveo | Anemia, Sickle Cell |
| 38 | B06AX03 | Voxelotor | Oxbryta | Anemia Anemia, Hemolytic Anemia, Sickle Cell |
| 39 | C01BB02 | Mexiletine hcl | Namuscla | Myotonic Disorders |
| 40 | C02KX04 | Macitentan | Opsumit | Hypertension, Pulmonary |
| 41 | C10AX18 | Volanesorsen | Waylivra | Hyperlipoproteinemia Type I |
| 42 | D02BB02 | Afamelanotide | Scenesse | Protoporphyria, Erythropoietic |
| 43 | D03BA03 | concentrate of proteolytic enzymes enriched in bromelain | NexoBrid | Debridement |
| 44 | H01AC07 | Somapacitan | Sogroya | Growth |
| 45 | H01AC08 | somatrogon | Ngenla | Growth and Development |
| 46 | H01AC09 | lonapegsomatropin | Lonapegsomatropin Ascendis Pharma | Growth and Development |
| 47 | H01CB05 | Pasireotide | Signifor | Acromegaly Pituitary ACTH Hypersecretion |
| 48 | H02CA02 | Osilodrostat | Isturisa (Insurisa) | Cushing Syndrome |
| 49 | H05AA03 | Parathyroid hormone | Natpar | Hypoparathyroidism |
| 50 | J01GB01 | Tobramycin | Tobi Podhaler | Cystic Fibrosis Respiratory Tract Infections |
| 51 | J01GB06 | Amikacin | Arikayce liposomal | Respiratory Tract Infections |
| 52 | J02AB02 | Ketoconazole | Ketoconazole HRA | Cushing Syndrome |
| 53 | J02AC | Isavuconazole | Cresemba | Aspergillosis |
| 54 | J04 | Pretomanid | Dovprela | Tuberculosis, Multidrug-Resistant |
| 55 | J04AA01 | Para-aminosalicylic acid | Granupas | Tuberculosis |
| 56 | J04AK05 | Bedaquiline | Sirturo | Tuberculosis, Multidrug-Resistant |
| 57 | J04AK06 | Delamanid | Deltyba | Tuberculosis, Multidrug-Resistant |
| 58 | J05 | Letermovir | Prevymis | Cytomegalovirus Infections |
| 59 | J05A | Bulevirtide | Hepcludex | Hepatitis D, Chronic |
| 60 | J06BB22 | Obiltoxaximab | Obiltoxaximab SFL (Nyxthracis ) | Anthrax |
| 61 | L01 | idecabtagene vicleucel | Abecma | Multiple Myeloma |
| 62 | L01 | tebentafusp | Kimmtrak | Uveal Neoplasms |
| 63 | L01 | Tisagenlecleucel | Kymriah | Precursor B-Cell Lymphoblastic Leukemia-Lymphoma Lymphoma, Large B-Cell, Diffuse |
| 64 | L01 | ripretinib | Qinlock | Gastrointestinal Stromal Tumors |
| 65 | L01AA05 | Chlormethine | Ledaga | Mycosis Fungoides |
| 66 | L01AB02 | Treosulfan | Trecondi | Hematopoietic Stem Cell Transplantation |
| 67 | L01BC08 | Decitabine | Dacogen | Leukemia, Myeloid |
| 68 | L01EE04 | selumetinib | Koselugo | Neurofibromatosis 1 |
| 69 | L01EJ02 | Fedratinib | Inrebic | Myeloproliferative Disorders; Primary Myelofibrosis |
| 70 | L01EN02 | Pemigatinib | Pemazyre | Cholangiocarcinoma |
| 71 | L01EX18 | Avapritinib | Ayvakyt | Gastrointestinal Stromal Tumors |
| 72 | L01FX06 | mosunetuzumab | Lunsumio | Lymphoma, Follicular |
| 73 | L01FX06 | Dinutuximab beta | Qarziba | Neuroblastoma |
| 74 | L01FX12 | tafasitamab | Minjuvi | Lymphoma, Large B-Cell, Diffuse |
| 75 | L01X | Autologous peripheral blood T cells CD4 and CD8 selected and CD3 and CD28 activated transduced with retroviral vector expressing anti-CD19 CD28/CD3-zeta chimeric antigen receptor and cultured | Tecartus | Lymphoma, Mantle-Cell |
| 76 | L01X | Axicabtagene ciloleucel | Yescarta | Lymphoma, Follicular Lymphoma, Large B-Cell, Diffuse |
| 77 | L01XC | Inotuzumab ozogamicin | Besponsa | Precursor Cell Lymphoblastic Leukemia-Lymphoma |
| 78 | L01XC | Blinatumomab | Blincyto | Precursor Cell Lymphoblastic Leukemia-Lymphoma |
| 79 | L01XC | Polatuzumab vedotin | Polivy | Lymphoma, B-Cell |
| 80 | L01XC05 | Gemtuzumab ozogamicin | Mylotarg | Leukemia, Myeloid, Acute |
| 81 | L01XC12 | Brentuximab vedotin | Adcetris | Lymphoma, Non-Hodgkin |
| 82 | L01XC15 | Obinutuzumab | Gazyvaro | Leukemia, Lymphocytic, Chronic, B-Cell |
| 83 | L01XC24 | Daratumumab | Darzalex | Multiple Myeloma |
| 84 | L01XC25 | Mogamulizumab | Poteligeo | Sezary Syndrome Mycosis Fungoides |
| 85 | L01XC39 | Belantamab mafodotin | Blenrep | Multiple Myeloma |
| 86 | L01XE | Cabozantinib | Cometriq | Thyroid Neoplasms |
| 87 | L01XE | Midostaurin | Rydapt | Leukemia, Myeloid, Acute Mastocytosis |
| 88 | L01XE05 | Sorafenib | Nexavar | Carcinoma, Hepatocellular Carcinoma, Renal Cell |
| 89 | L01XE10 | Everolimus | Votubia | Tuberous Sclerosis |
| 90 | L01XE24 | Ponatinib | Iclusig | Leukemia, Myeloid Leukemia, Lymphoid |
| 91 | L01XE54 | Gilteritinib | Xospata | Leukemia, Myeloid, Acute |
| 92 | L01XL05 | ciltacabtagene autoleucel | Carvykti | ciltacabtagene autoleucel |
| 93 | L01XX | Niraparib | Zejula | Fallopian Tube Neoplasms Peritoneal Neoplasms Ovarian Neoplasms |
| 94 | L01XX19 | irinotecan hydrochloride trihydrate | Onivyde pegylated liposomal | Pancreatic Neoplasms |
| 95 | L01XX42 | Panobinostat | Farydak | Multiple Myeloma |
| 96 | L01XX45 | Carfilzomib | Kyprolis | Multiple Myeloma |
| 97 | L01XX50 | Ixazomib | Ninlaro | Multiple Myeloma |
| 98 | L01XX63 | Glasdegib | Daurismo | Leukemia, Myeloid, Acute |
| 99 | L01XX67 | Tagraxofusp | Elzonris | Lymphoma |
| 100 | L01XY01 | Daunorubicin, cytarabine | Vyxeos liposomal | Leukemia, Myeloid, Acute |
| 101 | L03 | autologous CD34+ enriched cell fraction that contains CD34+ cells transduced with retroviral vector that encodes for the human ADA cDNA sequence | Strimvelis | Severe Combined Immunodeficiency |
| 102 | L04 | Darvadstrocel | Alofisel | Rectal Fistula in Crohn’s disease |
| 103 | L04 | pegcetacoplan | Aspaveli | Hemoglobinuria, Paroxysmal |
| 104 | L04 | avacopan | Tavneos | Microscopic Polyangiitis Wegener Granulomatosis |
| 105 | L04AA | Imlifidase | Idefirix | Desensitization, Immunologic; Kidney Transplantation |
| 106 | L04AA25 | Eculizumab | Soliris | Hemoglobinuria, Paroxysmal |
| 107 | L04AC | Satralizumab | Enspryng | Neuromyelitis Optica |
| 108 | L04AC11 | Siltuximab | Sylvant | Giant Lymph Node Hyperplasia |
| 109 | L04AX06 | Pomalidomide | Imnovid | Multiple Myeloma |
| 110 | M05BX | vosoritide | Voxzogo | Achondroplasia |
| 111 | M05BX05 | Burosumab | Crysvita | Hypophosphatemia, Familial Hypophosphatemic Rickets, X-Linked Dominan |
| 112 | M09 | Nusinersen | Spinraza | Muscular Atrophy, Spinal |
| 113 | M09AX03 | Ataluren | Translarna | Muscular Dystrophy, Duchenne |
| 114 | M09AX09 | onasemnogene abeparvovec | Zolgensma | Muscular Atrophy, Spinal |
| 115 | M09AX10 | Risdiplam | Evrysdi | Muscular Atrophy, Spinal |
| 116 | M09AX10 | birch bark extract | Filsuvez | Epidermolysis Bullosa Dystrophica Epidermolysis Bullosa, Junctional |
| 117 | N03 | Fenfluramine | Fintepla | Epilepsies, Myoclonic |
| 118 | N03AX | Cannabidiol | Epidyolex | Lennox Gastaut Syndrome Epilepsies, Myoclonic |
| 119 | N05CH | Tasimelteon | Hetlioz | Sleep Disorders, Circadian Rhythm |
| 120 | N06BX13 | Idebenone | Raxone | Optic Atrophy, Hereditary, Leber |
| 121 | N07 | Autologous CD34+ cells encoding ARSA gene | Libmeldy | Leukodystrophy, Metachromatic |
| 122 | N07 | Patisiran | Onpattro | Amyloidosis, Familial |
| 123 | N07 | Inotersen | Tegsedi | Amyloidosis |
| 124 | N07XX08 | Tafamidis | Vyndaqel | Amyloidosis |
| 125 | N07XX11 | Pitolisant | Wakix | Narcolepsy |
| 126 | P01BE03 | artesunate | Artesunate Amivas | Malaria |
| 127 | R07AX31 | Tezacaftor, Ivacaftor | Symkevi | Cystic Fibrosis |
| 128 | R07AX32 | Ivacaftor, tezacaftor, elexacaftor | Kaftrio | Cystic Fibrosis |
| 129 | S01 | cenegermin | Oxervate | Keratitis |
| 130 | S01XA18 | Ciclosporin | Verkazia | Conjunctivitis Keratitis |
| 131 | S01XA19 | Ex vivo expanded autologous human corneal epithelial cells containing stem cells | Holoclar | Stem Cell Transplantation Corneal Diseases |
| 132 | S01XA21 | Mercaptamine | Cystadrops | Cystinosis |
| 133 | S01XA27 | Voretigene neparvovec | Luxturna | Leber Congenital Amaurosis, Retinitis Pigmentosa |
| 134 | V03AF09 | glucarpidase | Voraxaze | Metabolic Side Effects of Drugs and Substances |
| 135 | V09IX | Edotreotide | SomaKit TOC | Neuroendocrine Tumors Radionuclide Imaging |
| 136 | V10XX04 | Lutetium (177Lu) oxodotreotide | Lutathera | Neuroendocrine Tumors |

###### Appendix 2 (Table) Frequency of positive recommendation by a particular ODs in different countries

| **ATC classification** | **International non-proprietary name (INN) or common name** | **Trade name** | **Number of countries where the OD got at least one positive recommendation** | **% of countries** |
| --- | --- | --- | --- | --- |
| **A16AX** | **Migalastat** | **Galafold** | **10** | **83%** |
| **C02KX04** | **Macitentan** | **Opsumit** | **10** | **83%** |
| **L01XE** | **Cabozantinib** | **Rydapt** | **10** | **83%** |
| **L01XE05** | **Sorafenib** | **Nexavar** | **10** | **83%** |
| **L04AX06** | **Pomalidomide** | **Imnovid** | **10** | **83%** |
| A07EA06 | Budesonide | Jorveza | 9 | 75% |
| A16AX10 | Givosiran | Cerdelga | 9 | 75% |
| J01GB01 | Tobramycin | Tobi Podhaler | 9 | 75% |
| L01X | Autologous peripheral blood T cells CD4 and CD8 selected and CD3 and CD28 activated transduced with retroviral vector expressing anti-CD19 CD28/CD3-zeta chimeric antigen receptor and cultured | Yescarta | 9 | 75% |
| L01XC | Inotuzumab ozogamicin | Blincyto | 9 | 75% |
| L01XC12 | Brentuximab vedotin | Adcetris | 9 | 75% |
| L01XC24 | Daratumumab | Darzalex | 9 | 75% |
| L01XX | Niraparib | Zejula | 9 | 75% |
| L01XX45 | Carfilzomib | Kyprolis | 9 | 75% |
| M09AX09 | onasemnogene abeparvovec | Zolgensma | 9 | 75% |
| M09AX10 | Risdiplam | Evrysdi | 9 | 75% |
| N07XX08 | Tafamidis | Vyndaqel | 9 | 75% |
| R07AX32 | Ivacaftor, tezacaftor, elexacaftor | Kaftrio | 9 | 75% |
| H01CB05 | Pasireotide | Signifor | 8 | 67% |
| J02AC | Isavuconazole | Cresemba | 8 | 67% |
| J05 | Letermovir | Prevymis | 8 | 67% |
| L01 | idecabtagene vicleucel | Kymriah | 8 | 67% |
| L01XC | Blinatumomab | Besponsa | 8 | 67% |
| L01XC05 | Gemtuzumab ozogamicin | Mylotarg | 8 | 67% |
| L01XE10 | Everolimus | Votubia | 8 | 67% |
| L01XE24 | Ponatinib | Iclusig | 8 | 67% |
| L01XE54 | Gilteritinib | Xospata | 8 | 67% |
| M09 | Nusinersen | Spinraza | 8 | 67% |
| N03AX | Cannabidiol | Epidyolex | 8 | 67% |
| B01A | Caplacizumab | Cablivi | 7 | 58% |
| B02BD04 | Eftrenonacog alfa | Alprolix | 7 | 58% |
| B06AC05 | Lanadelumab | Takhzyro | 7 | 58% |
| L01XC15 | Obinutuzumab | Gazyvaro | 7 | 58% |
| L01XX50 | Ixazomib | Ninlaro | 7 | 58% |
| N06BX13 | Idebenone | Raxone | 7 | 58% |
| N07 | Autologous CD34+ cells encoding ARSA gene | Onpattro | 7 | 58% |
| N07XX11 | Pitolisant | Wakix | 7 | 58% |
| R07AX31 | Tezacaftor, Ivacaftor | Symkevi | 7 | 58% |
| A05AA04 | Obeticholic acid | Ocaliva | 6 | 50% |
| A16AB10 | Velaglucerase alfa | Vpriv | 6 | 50% |
| A16AX08 | Teduglutide | Revestive | 6 | 50% |
| H01AC08 | somatrogon | Ngenla | 6 | 50% |
| H05AA03 | Parathyroid hormone | Natpar | 6 | 50% |
| L01BC08 | Decitabine | Dacogen | 6 | 50% |
| L01EJ02 | Fedratinib | Inrebic | 6 | 50% |
| L01FX06 | mosunetuzumab | Qarziba | 6 | 50% |
| L01X | Axicabtagene ciloleucel | Tecartus | 6 | 50% |
| L01XC | Polatuzumab vedotin | Polivy | 6 | 50% |
| L01XE | Midostaurin | Cometriq | 6 | 50% |
| L01XX42 | Panobinostat | Farydak | 6 | 50% |
| L01XY01 | Daunorubicin, cytarabine | Vyxeos liposomal | 6 | 50% |
| L04 | Darvadstrocel | Aspaveli | 6 | 50% |
| L04AA25 | Eculizumab | Soliris | 6 | 50% |
| M05BX05 | Burosumab | Crysvita | 6 | 50% |
| N07 | Patisiran | Tegsedi | 6 | 50% |
| S01XA21 | Mercaptamine | Cystadrops | 6 | 50% |
| S01XA27 | Voretigene neparvovec | Luxturna | 6 | 50% |
| A05AA01 | Chenodeoxycholic acid | Chenodeoxycholic acid Leadiant | 5 | 42% |
| A16A | telotristat ethyl | Xermelo | 5 | 42% |
| A16AA04 | Mercaptamine | Procysbi | 5 | 42% |
| A16AB | Cerliponase alfa | Strensiq | 5 | 42% |
| A16AX1 | Eliglustat | Givlaari | 5 | 42% |
| A16AX18 | Lumasiran | Oxlumo | 5 | 42% |
| B03XA06 | Luspatercept | Reblozyl | 5 | 42% |
| B06AX01 | Crizanlizumab | Adakveo | 5 | 42% |
| C10AX18 | Volanesorsen | Waylivra | 5 | 42% |
| L01AA05 | Chlormethine | Ledaga | 5 | 42% |
| L01XC25 | Mogamulizumab | Poteligeo | 5 | 42% |
| L01XX19 | irinotecan hydrochloride trihydrate | Onivyde pegylated liposomal | 5 | 42% |
| L04AA | Imlifidase | Idefirix | 5 | 42% |
| L04AC11 | Siltuximab | Sylvant | 5 | 42% |
| S01XA18 | Ciclosporin | Verkazia | 5 | 42% |
| S01XA19 | Ex vivo expanded autologous human corneal epithelial cells containing stem cells | Holoclar | 5 | 42% |
| V10XX04 | Lutetium (177Lu) oxodotreotide | Lutathera | 5 | 42% |
| A08AA | Setmelanotide | Imcivree | 4 | 33% |
| A16AA | Metreleptin | Myalepta | 4 | 33% |
| A16AB | Asfotase alfa | Brineura | 4 | 33% |
| A16AB15 | Velmanase alfa | Lamzede | 4 | 33% |
| A16AX09 | Glycerol phenylbutyrate | Ravicti | 4 | 33% |
| B01AX01 | Defibrotide | Defitelio | 4 | 33% |
| B02BD04 | Albutrepenonacog alfa | Idelvion | 4 | 33% |
| H02CA02 | Osilodrostat | Isturisa (Insurisa) | 4 | 33% |
| J01GB06 | Amikacin | Arikayce liposomal | 4 | 33% |
| J02AB02 | Ketoconazole | Ketoconazole HRA | 4 | 33% |
| J04AK05 | Bedaquiline | Sirturo | 4 | 33% |
| L01EN02 | Pemigatinib | Pemazyre | 4 | 33% |
| L01XC39 | Belantamab mafodotin | Blenrep | 4 | 33% |
| M09AX03 | Ataluren | Translarna | 4 | 33% |
| A16 | Sebelipase alfa | Kanuma | 3 | 25% |
| A16AB12 | elosulfase alfa | Vimizim | 3 | 25% |
| B01AC21 | Treprostinil | Trepulmix | 3 | 25% |
| C01BB02 | Mexiletine hcl | Namuscla | 3 | 25% |
| D02BB02 | Afamelanotide | Scenesse | 3 | 25% |
| J04AK06 | Delamanid | Deltyba | 3 | 25% |
| J05A | Bulevirtide | Hepcludex | 3 | 25% |
| L01AB02 | Treosulfan | Trecondi | 3 | 25% |
| L01EX18 | Avapritinib | Ayvakyt | 3 | 25% |
| L01FX12 | tafasitamab | Minjuvi | 3 | 25% |
| L01XL05 | ciltacabtagene autoleucel | Carvykti | 3 | 25% |
| L01XX63 | Glasdegib | Daurismo | 3 | 25% |
| L04 | pegcetacoplan | Alofisel | 3 | 25% |
| L04AC | Satralizumab | Enspryng | 3 | 25% |
| M05BX | vosoritide | Voxzogo | 3 | 25% |
| N07 | Inotersen | Libmeldy | 3 | 25% |
| V03AF09 | glucarpidase | Voraxaze | 3 | 25% |
| A05AX | Odevixibat | Bylvay | 2 | 17% |
| A07EA06 | budesonide | Kinpeygo | 2 | 17% |
| A16AB18 | Vestronidase alfa | Mepsevii | 2 | 17% |
| A16AB19 | Pegvaliase | Palynziq | 2 | 17% |
| D03BA03 | concentrate of proteolytic enzymes enriched in bromelain | NexoBrid | 2 | 17% |
| H01AC07 | Somapacitan | Sogroya | 2 | 17% |
| L01 | tebentafusp | Kimmtrak | 2 | 17% |
| L03 | autologous CD34+ enriched cell fraction that contains CD34+ cells transduced with retroviral vector that encodes for the human ADA cDNA sequence | Strimvelis | 2 | 17% |
| N03 | Fenfluramine | Fintepla | 2 | 17% |
| S01 | cenegermin | Oxervate | 2 | 17% |
| V09IX | Edotreotide | SomaKit TOC | 2 | 17% |
| A05AA03 | Cholic acid | Orphacol | 1 | 8% |
| A10BB01 | Glibenclamide | Amglidia | 1 | 8% |
| A16AB25 | olipudase alfa | Xenpozyme | 1 | 8% |
| A16AB26 | eladocagene exuparvovec | Upstaza | 1 | 8% |
| A16AX20 | lonafarnib | Zokinvy | 1 | 8% |
| B02BD13 | Human coagulation factor X | Coagadex | 1 | 8% |
| B06AX03 | Voxelotor | Oxbryta | 1 | 8% |
| J04 | Pretomanid | Dovprela | 1 | 8% |
| J04AA01 | Para-aminosalicylic acid | Granupas | 1 | 8% |
| L01 | Tisagenlecleucel | Abecma | 1 | 8% |
| L01 | ripretinib | Qinlock | 1 | 8% |
| L01EE04 | selumetinib | Koselugo | 1 | 8% |
| L01XX67 | Tagraxofusp | Elzonris | 1 | 8% |
| L04 | avacopan | Tavneos | 1 | 8% |
| M09AX10 | birch bark extract | Filsuvez | 1 | 8% |
| P01BE03 | artesunate | Artesunate Amivas | 1 | 8% |
| H01AC09 | lonapegsomatropin | Lonapegsomatropin Ascendis Pharma | 0 | 0% |
| J06BB22 | Obiltoxaximab | Obiltoxaximab SFL (Nyxthracis ) | 0 | 0% |
| L01FX06 | Dinutuximab beta | Lunsumio | 0 | 0% |
| N05CH | Tasimelteon | Hetlioz | 0 | 0% |

###### Appendix 3 (Table) Frequency of reimbursement of a particular ODs in different countries

| **ATC classification** | **International non-proprietary name (INN) or common name** | **Trade name** | **Number of countries where the OD is reimbursed for at least one indication** | **% of countries** |
| --- | --- | --- | --- | --- |
| **C02KX04** | **Macitentan** | **Opsumit** | **12** | **100%** |
| L01XC05 | Gemtuzumab ozogamicin | Mylotarg | 11 | 92% |
| L01XC24 | Daratumumab | Darzalex | 11 | 92% |
| L01XE | Cabozantinib | Rydapt | 11 | 92% |
| L01XE05 | Sorafenib | Nexavar | 11 | 92% |
| L01XX45 | Carfilzomib | Kyprolis | 11 | 92% |
| L04AX06 | Pomalidomide | Imnovid | 11 | 92% |
| A16AX | Migalastat | Galafold | 10 | 83% |
| A16AX10 | Givosiran | Cerdelga | 10 | 83% |
| H01CB05 | Pasireotide | Signifor | 10 | 83% |
| L01 | idecabtagene vicleucel | Kymriah | 10 | 83% |
| L01XC12 | Brentuximab vedotin | Adcetris | 10 | 83% |
| L01XC15 | Obinutuzumab | Gazyvaro | 10 | 83% |
| L01XE10 | Everolimus | Votubia | 10 | 83% |
| L01XE24 | Ponatinib | Iclusig | 10 | 83% |
| L01XE54 | Gilteritinib | Xospata | 10 | 83% |
| L01XX | Niraparib | Zejula | 10 | 83% |
| M09 | Nusinersen | Spinraza | 10 | 83% |
| N07XX08 | Tafamidis | Vyndaqel | 10 | 83% |
| R07AX32 | Ivacaftor, tezacaftor, elexacaftor | Kaftrio | 10 | 83% |
| A07EA06 | Budesonide | Jorveza | 9 | 75% |
| A16AB10 | Velaglucerase alfa | Vpriv | 9 | 75% |
| A16AX08 | Teduglutide | Revestive | 9 | 75% |
| B02BD04 | Eftrenonacog alfa | Alprolix | 9 | 75% |
| B06AC05 | Lanadelumab | Takhzyro | 9 | 75% |
| J02AC | Isavuconazole | Cresemba | 9 | 75% |
| J05 | Letermovir | Prevymis | 9 | 75% |
| L01X | Autologous peripheral blood T cells CD4 and CD8 selected and CD3 and CD28 activated transduced with retroviral vector expressing anti-CD19 CD28/CD3-zeta chimeric antigen receptor and cultured | Yescarta | 9 | 75% |
| L01XC | Inotuzumab ozogamicin | Besponsa | 9 | 75% |
| L01XC | Blinatumomab | Blincyto | 9 | 75% |
| L01XC | Polatuzumab vedotin | Polivy | 9 | 75% |
| L01XX50 | Ixazomib | Ninlaro | 9 | 75% |
| L04AA | Imlifidase | Idefirix | 9 | 75% |
| L04AA25 | Eculizumab | Soliris | 9 | 75% |
| M09AX09 | onasemnogene abeparvovec | Zolgensma | 9 | 75% |
| M09AX10 | Risdiplam | Evrysdi | 9 | 75% |
| R07AX31 | Tezacaftor, Ivacaftor | Symkevi | 9 | 75% |
| B01A | Caplacizumab | Cablivi | 8 | 67% |
| B03XA06 | Luspatercept | Reblozyl | 8 | 67% |
| H01AC08 | somatrogon | Ngenla | 8 | 67% |
| J01GB01 | Tobramycin | Tobi Podhaler | 8 | 67% |
| L01FX06 | mosunetuzumab | Qarziba | 8 | 67% |
| N03AX | Cannabidiol | Epidyolex | 8 | 67% |
| N06BX13 | Idebenone | Raxone | 8 | 67% |
| N07 | Autologous CD34+ cells encoding ARSA gene | Onpattro | 8 | 67% |
| S01XA27 | Voretigene neparvovec | Luxturna | 8 | 67% |
| V10XX04 | Lutetium (177Lu) oxodotreotide | Lutathera | 8 | 67% |
| A05AA04 | Obeticholic acid | Ocaliva | 7 | 58% |
| A16AX09 | Glycerol phenylbutyrate | Ravicti | 7 | 58% |
| A16AX1 | Eliglustat | Givlaari | 7 | 58% |
| A16AX18 | Lumasiran | Oxlumo | 7 | 58% |
| L01EJ02 | Fedratinib | Inrebic | 7 | 58% |
| L01XC25 | Mogamulizumab | Poteligeo | 7 | 58% |
| L01XX42 | Panobinostat | Farydak | 7 | 58% |
| L01XY01 | Daunorubicin, cytarabine | Vyxeos liposomal | 7 | 58% |
| L04 | Darvadstrocel | Aspaveli | 7 | 58% |
| M05BX05 | Burosumab | Crysvita | 7 | 58% |
| N07 | Patisiran | Tegsedi | 7 | 58% |
| A08AA | Setmelanotide | Imcivree | 6 | 50% |
| A16AA04 | Mercaptamine | Procysbi | 6 | 50% |
| B01AC21 | Treprostinil | Trepulmix | 6 | 50% |
| B02BD04 | Albutrepenonacog alfa | Idelvion | 6 | 50% |
| B06AX01 | Crizanlizumab | Adakveo | 6 | 50% |
| J04AK05 | Bedaquiline | Sirturo | 6 | 50% |
| L01AA05 | Chlormethine | Ledaga | 6 | 50% |
| L01BC08 | Decitabine | Dacogen | 6 | 50% |
| L01X | Axicabtagene ciloleucel | Tecartus | 6 | 50% |
| L01XC39 | Belantamab mafodotin | Blenrep | 6 | 50% |
| L04AC | Satralizumab | Enspryng | 6 | 50% |
| L04AC11 | Siltuximab | Sylvant | 6 | 50% |
| S01XA19 | Ex vivo expanded autologous human corneal epithelial cells containing stem cells | Holoclar | 6 | 50% |
| A16 | Sebelipase alfa | Kanuma | 5 | 42% |
| A16A | telotristat ethyl | Xermelo | 5 | 42% |
| H02CA02 | Osilodrostat | Isturisa (Insurisa) | 5 | 42% |
| J02AB02 | Ketoconazole | Ketoconazole HRA | 5 | 42% |
| L01EN02 | Pemigatinib | Pemazyre | 5 | 42% |
| L01FX12 | tafasitamab | Minjuvi | 5 | 42% |
| L01XE | Midostaurin | Cometriq | 5 | 42% |
| L01XX19 | irinotecan hydrochloride trihydrate | Onivyde pegylated liposomal | 5 | 42% |
| M05BX | vosoritide | Voxzogo | 5 | 42% |
| M09AX03 | Ataluren | Translarna | 5 | 42% |
| N03 | Fenfluramine | Fintepla | 5 | 42% |
| N07XX11 | Pitolisant | Wakix | 5 | 42% |
| S01XA21 | Mercaptamine | Cystadrops | 5 | 42% |
| A16AA | Metreleptin | Myalepta | 4 | 33% |
| A16AB | Cerliponase alfa | Brineura | 4 | 33% |
| A16AB12 | elosulfase alfa | Vimizim | 4 | 33% |
| A16AB15 | Velmanase alfa | Lamzede | 4 | 33% |
| B01AX01 | Defibrotide | Defitelio | 4 | 33% |
| C01BB02 | Mexiletine hcl | Namuscla | 4 | 33% |
| C10AX18 | Volanesorsen | Waylivra | 4 | 33% |
| D03BA03 | concentrate of proteolytic enzymes enriched in bromelain | NexoBrid | 4 | 33% |
| H05AA03 | Parathyroid hormone | Natpar | 4 | 33% |
| J01GB06 | Amikacin | Arikayce liposomal | 4 | 33% |
| J04AK06 | Delamanid | Deltyba | 4 | 33% |
| J05A | Bulevirtide | Hepcludex | 4 | 33% |
| L01 | tebentafusp | Kimmtrak | 4 | 33% |
| L01AB02 | Treosulfan | Trecondi | 4 | 33% |
| L01EX18 | Avapritinib | Ayvakyt | 4 | 33% |
| L03 | autologous CD34+ enriched cell fraction that contains CD34+ cells transduced with retroviral vector that encodes for the human ADA cDNA sequence | Strimvelis | 4 | 33% |
| L04 | pegcetacoplan | Alofisel | 4 | 33% |
| N07 | Inotersen | Libmeldy | 4 | 33% |
| S01XA18 | Ciclosporin | Verkazia | 4 | 33% |
| V03AF09 | glucarpidase | Voraxaze | 4 | 33% |
| A05AA01 | Chenodeoxycholic acid | Chenodeoxycholic acid Leadiant | 3 | 25% |
| A05AA03 | Cholic acid | Orphacol | 3 | 25% |
| A05AX | Odevixibat | Bylvay | 3 | 25% |
| A16AB | Asfotase alfa | Strensiq | 3 | 25% |
| A16AB18 | Vestronidase alfa | Mepsevii | 3 | 25% |
| A16AB19 | Pegvaliase | Palynziq | 3 | 25% |
| B06AX03 | Voxelotor | Oxbryta | 3 | 25% |
| D02BB02 | Afamelanotide | Scenesse | 3 | 25% |
| L01EE04 | selumetinib | Koselugo | 3 | 25% |
| L01XX63 | Glasdegib | Daurismo | 3 | 25% |
| L01XX67 | Tagraxofusp | Elzonris | 3 | 25% |
| V09IX | Edotreotide | SomaKit TOC | 3 | 25% |
| A10BB01 | Glibenclamide | Amglidia | 2 | 17% |
| A16AB26 | eladocagene exuparvovec | Upstaza | 2 | 17% |
| B02BD13 | Human coagulation factor X | Coagadex | 2 | 17% |
| J04 | Pretomanid | Dovprela | 2 | 17% |
| J04AA01 | Para-aminosalicylic acid | Granupas | 2 | 17% |
| L01 | Tisagenlecleucel | Abecma | 2 | 17% |
| L01 | ripretinib | Qinlock | 2 | 17% |
| L01XL05 | ciltacabtagene autoleucel | Carvykti | 2 | 17% |
| L04 | avacopan | Tavneos | 2 | 17% |
| A07EA06 | budesonide | Kinpeygo | 1 | 8% |
| A16AB25 | olipudase alfa | Xenpozyme | 1 | 8% |
| A16AX20 | lonafarnib | Zokinvy | 1 | 8% |
| L01FX06 | Dinutuximab beta | Lunsumio | 1 | 8% |
| M09AX10 | birch bark extract | Filsuvez | 1 | 8% |
| N05CH | Tasimelteon | Hetlioz | 1 | 8% |
| P01BE03 | artesunate | Artesunate Amivas | 1 | 8% |
| H01AC07 | Somapacitan | Sogroya | 0 | 0% |
| H01AC09 | lonapegsomatropin | Lonapegsomatropin Ascendis Pharma | 0 | 0% |
| J06BB22 | Obiltoxaximab | Obiltoxaximab SFL (Nyxthracis ) | 0 | 0% |
| S01 | cenegermin | Oxervate | 0 | 0% |
